# Alpha synuclein-mediated cytoskeletal dysfunction impairs myelination in human oligodendrocytes

**DOI:** 10.1007/s00401-025-02933-z

**Published:** 2025-09-19

**Authors:** Jeanette Wihan, Kristina Battis, Alana Hoffmann, Farina Windener, Marcus Himmler, Anish Varghese, Aron Koller, Isabell Karnatz, Dirk W. Schubert, Friederike Zunke, Wei Xiang, Tanja Kuhlmann, Jürgen Winkler

**Affiliations:** 1 Division of Molecular Neurology, University Hospital Erlangen, Friedrich-Alexander-University (FAU) Erlangen-Nürnberg, 91054 Erlangen, Germany; 2 Project Center for Stem Cell Process Engineering, Fraunhofer Institute for Biomedical Engineering (IBMT), 97070 Würzburg, Germany; 3 Keenan Research Centre for Biomedical Science and Barlo Multiple Sclerosis Centre, St Michael’s Hospital, Toronto, ON Canada; 4 Department of Immunology, The University of Toronto, Toronto, ON Canada; 5 Institute of Neuropathology, University Hospital Münster, 48149 Muenster, Germany; 6 Department of Materials Science and Engineering, Institute of Polymer Materials, Friedrich-Alexander-University (FAU) Erlangen-Nürnberg, 91058 Erlangen, Germany; 7 Department of Molecular and Cellular Biotechnology, Saarland University, 66123 Saarbrücken, Germany

**Keywords:** Alpha synuclein, Human oligodendrocytes, Multiple system atrophy, Myelin, Actin

## Abstract

Oligodendroglial alpha-synuclein (aSyn) deposits are a key feature in the atypical parkinsonian disorder, multiple system atrophy (MSA) linked to profound myelin loss and neurodegeneration while precise cellular and molecular mechanisms remain unclear. We generated human oligodendrocytes (hOLs) from induced pluripotent stem cells to investigate the impact of aSyn on oligodendroglial morphology, differentiation, and function. We observed an aSyn-induced myelinogenic dysfunction characterized by impaired oligodendroglial process outgrowth, altered cell shape, and increased perinuclear accumulation of the tubulin polymerization promoting protein TPPP/p25α. These changes were associated with a reduced capacity to ensheath axons and were linked to compromised actin remodeling machinery. Actin imbalances were confirmed in *post-mortem* putaminal tissue from MSA patients. Treatment with a rho-associated protein kinase inhibitor rescued oligodendroglial process formation and improved ensheathment in aSyn-expressing hOLs. Our work emphasizes the aSyn-mediated interference with actin dynamics as a key pathogenic mechanism in MSA, pointing toward a novel therapeutic target for improving myelin maintenance.

**Supplementary Information:**

The online version contains supplementary material available at 10.1007/s00401-025-02933-z.

## Introduction

Multiple system atrophy (MSA) is a rare and rapidly progressive oligodendroglial synucleinopathy. It is a primarily sporadic disease characterized by severe myelin loss with secondary, widespread degeneration of neurons in multiple central nervous system (CNS) regions including the nigrostriatal system, cerebellum, pons, and inferior olives. The neurodegenerative processes are accompanied by a profound astro- and microgliosis leading to severe autonomic dysfunctions, parkinsonism, and cerebellar ataxia [12]. In the absence of a causal treatment to slow or halt the fatal progression of the disease, current therapeutic approaches are limited to the symptomatic treatment of motor and non-motor symptoms. The main neuropathologic hallmark of the disease is the accumulation of alpha-synuclein (aSyn) within oligodendrocytes forming glial cytoplasmic inclusions (GCIs) [32, 33]. Originally discovered as a presynaptic protein in neurons, aSyn, encoded by the *SNCA* gene, has also been detected in isolated oligodendrocytes derived from neonatal wild-type mice and brain tissue of MSA patients [2, 7].

Oligodendrocytes are developmental derivatives of neural stem cells of the brain and spinal cord [20, 40, 58]. After migration and dispersion throughout the CNS, oligodendrocyte precursor cells (OPCs) extend multiple cellular processes to explore their microenvironment, which ultimately evolve into large, flat membrane sheaths enwrapping axons [48, 51]. The precise orchestration of these substantial structural changes demands a highly dynamic cytoskeletal machinery. In the early stages of process extension, actin filament assembly is critical and contributes to the formation of the growth cone at the tip of oligodendrocyte processes. As the process progresses, actin depolymerization becomes essential to facilitate myelin formation and compaction [28, 66].This dynamic rearrangement of the cytoskeleton is reflected in gene profiling studies of primary rodent OPCs and isolated oligodendroglial lineage cells demonstrating enriched transcripts for actin assembly and disassembly factors in premature and myelinating oligodendrocytes, respectively [65, 66]. Moreover, impaired actin dynamics in the CNS have been linked to hypomyelination and motor dysfunctions resulting in tremor and ataxia [28, 66]. These findings indicate a critical role of actin dynamics in myelin formation and, consequently, proper brain function, but little attention has been drawn to the oligodendroglial cytoskeleton and morphology in MSA.

The precise pathomechanisms underlying MSA remain elusive, however, accumulating evidence indicates that the intraoligodendroglial presence of distinct aSyn species is pivotal in MSA pathogenesis [18, 33, 53]. We, therefore, aimed to characterize effects of aSyn on oligodendroglial morphology, differentiation, and function using human induced pluripotent stem cells (hiPSCs). Here, we present an aSyn-induced myelinogenic dysfunction, which is linked to an impaired outgrowth of oligodendroglial processes in differentiating human oligodendrocytes (hOLs). Phenotypic changes in shape and appearance were observed in aSyn-expressing hOLs, accompanied by increased perinuclear accumulation of TPPP and defects in actin remodeling. Consistently, we confirmed actin imbalances in *post-mortem* putaminal tissue derived from MSA patients. Importantly, interfering with actin remodeling through the rho-associated protein kinase inhibitor (RI) Y-27632 accelerated the formation of oligodendroglial processes and improved the capacity of hOLs to ensheath axon-mimicking structures in vitro. Thus, the present work underlines the importance of actin dynamics in human myelination and suggests a pathogenic link between an increased intraoligodendroglial aSyn level and the dynamics of actin remodeling for process outgrowth.

## Methods

### Human *post-mortem* tissue

Frozen putaminal *post-mortem* tissue of five MSA patients and controls was obtained from the Netherlands Brain Bank (NBB), Netherlands Institute for Neuroscience, Amsterdam (open access: http://www.brainbank.nl). MSA patients were clinically and neuropathologically diagnosed according to current consensus guidelines. Absence of neurological diseases and aSyn pathology was prerequisite for age-and sex-matched controls. For histological analyses, tissue was sectioned in 6 µm cryosections prior to fixation and staining (Suppl. Table 1). Fresh frozen tissue was used for Western blot and reverse transcription quantitative PCR for gene expression analysis.

### hiPSC culture and genome-editing

Dermal fibroblasts were obtained from skin biopsies of two healthy donors (C1: male 71 years, C2: female 66 years) and reprogrammed using the Cytotune 2.0 Sendai kit (Thermo Fisher) to generate hiPSCs. All skin biopsy sampling procedures were performed at the Outpatient Center for Movement Disorder at the University Hospital Erlangen in accordance with the local Institutional Review Board approval (No. 259_17B, University Hospital Erlangen, Friedrich-Alexander-Universität Erlangen-Nürnberg, Erlangen, Germany). Written informed consents of donors are on file at the Division of Molecular Neurology, University Hospital Erlangen, Germany. hiPSCs were cultured on Matrigel (growth-factor reduced, Corning) at 37 °C in a humidified 5% CO_2_ incubator and passaged twice a week using Gentle Cell Dissociation Reagent. The control hiPSC lines UKERiO3H-S1-006 (C1) and UKERi82A-S1-005 (C2) were included in this study.

### Stable integration of SOX10, OLIG2, and NKX6.2 (SON) cassette in iPSCs

For the generation of inducible SON-iPSC lines, we adapted an one-in-all vector strategy for genomic integration to a safe genomic harbour as described earlier [15]. Human iPSCs were targeted with two zinc finger nucleases integrating the expression cassette containing the SON transcriptions factors [9] under control of a Tet-On inducible promotor (TRE3G), a reverse tetracycline transactivator (rtTA) under control of the CMV promoter as well as a puromycin resistance for selection, into the adeno-associated-virus-site 1 (AAVS1) locus on chromosome 19 [34, 50]. Surviving single cells were grown to colonies, picked, and analyzed via PCR for the correct and bi-allelic integration of the construct. Primer sequences are summarized in Suppl. Table 2.

### Generation of neural precursor cells (NPCs)

NPCs were derived from hiPSCs as previously described [41, 52]. After dual SMAD inhibition, NPCs were cultured in N2B27 medium consisting of 50% DMEM-F12 / GlutaMAX, 50% neurobasal medium, 1X B27 supplement (lacking vitamin A), 1X N2 supplement, and 1% penicillin/streptomycin (PS). N2B27 was further supplemented with 3 µM CHIR, 0.5 µM SAG, and 150 µM ascorbic acid (AA; NPC-medium, NPCM). Cultures were passaged once a week using accutase with medium changes every other day.

### Oligodendrocyte differentiation

NPCs were plated at a density of 26,300 cells/cm^2^ in NPCM supplemented with 3 µg/mL doxycycline (dox). RI Y-27632 dihydrochloride (2 µM, Enzo Life Sciences) was added for 24 h. Cells were transduced with EF-SNCA or EF- green fluorescent protein (GFP) lentiviral vector (generated as described below in “Generation of lentiviral particles”) using a multiplicity of infection of 1. Viral medium was removed after 24 h and replaced by glial induction medium (GIM) consisting of N2B27 (without neurobasal medium), 1 µM SAG, 10 ng/mL PDGF-AA, 10 ng/mL NT-3, 10 ng/ml IGF-1, 10 ng/mL T3, 200 µM AA, 0.1% Trace Elements B, and 3 µg/mL dox. GIM was changed every other day and replaced by differentiation medium (DM) supplemented with 3 µg/mL dox after four days. DM consists of N2B27 (without neurobasal medium), 10 ng/mL NT-3, 10 ng/mL IGF-1, 90 ng/mL T3, 200 µM AA, 0.1% Trace Elements B, and 100 µM db-cAMP. Seven days after glial induction, cells were detached using accutase and replated at a density of 26,300 cells/cm^2^ in DM supplemented with 3 µg/mL dox. RI (2 µM) was added for 24 h. From day 12 on, dox was withdrawn from the medium and cells were cultured in DM with media changes every other day.

### Nanofiber assay

Aligned polycaprolactone (PCL) nanofibers with a diameter of 700 nm were synthesized by electrospinning as previously described [19, 43], fixed onto tissue carriers (13 mm), and coated with Cy5-labeled poly-L-lysine (PLL, Nanocs) at a final concentration of 100 µg/mL at room temperature (RT) overnight followed by Matrigel coating. Differentiating hOLs were replated onto nanofibers in DM supplemented with RI (2 µM) at day 9 of differentiation. 100,000 cells were seeded per tissue carrier.

### Human triple-culture

First, Neurogenin 2 (NGN2)-inducible cortical neurons were derived from the hiPSC line BIONi010-C-13 (sample ID: SAMEA103988285), obtained from the European Bank for Induced Pluripotent Stem Cells (EBiSC), and generated as previously described [60]. NGN2 neurons were plated at a density of 10,000 cells/cm^2^ in neuronal basal medium (NBM) consisting of 50% DMEM-F12/GlutaMAX, 50% neurobasal medium, 0.5X B27 supplement, 0.5X N2 supplement, 0.5X GlutaMAX™ Supplement, 0.5X MEM Non-Essential Amino Acids solution, 0.5 mM sodium pyruvate, 50 µM 2-mercaptoethanol, 0.025% insulin solution human and 1% PS. NBM was further supplemented with 2 µg/mL dox. After 4 days of differentiation, human primary astrocytes (ScienCell, #1800) were plated at a density of 10,000 cells/cm^2^ onto the pre-differentiated neurons. Co-cultures of neurons and astrocytes were maintained using NBM + 20 ng/ mL GDNF and 20 ng/ mL BDNF for 2 weeks. Differentiating hOLs were replated onto these co-cultures in DM supplemented with 20 ng/mL GDNF, 20 ng/ mL BDNF, 3 µg/mL dox, and RI (2 µM) at day 9 of differentiation. 30,000 cells were seeded per 24-well. From day 12 on, dox was withdrawn from the medium and cells were cultured in DM supplemented with 20 ng/ mL GDNF, 20 ng/mL BDNF with media changes every other day. Human triple-cultures were fixed at day 23 of differentiation.

### Immunocytochemistry and imaging

Cells were sequentially fixed with 2% paraformaldehyde (PFA, 8 min, RT) in culture medium and thereafter with 4% PFA in PBS (10 min, RT). As an exception, O4-staining was performed prior to fixation (30 min, 37 °C). After fixation, cells were blocked and permeabilized with PBS/3% donkey serum/0.1% Triton X-100 for 1 h at RT, or with fish skin gelatin buffer (FSGB: tris-buffered saline with 0.4% cold water fish skin gelatin in water, 1% bovine serum albumin (BSA), and 0.1% Triton X-100) for 30 min at RT. For tubulin polymerization promoting protein TPPP/p25α (TPPP) immunostaining, FSGB containing 0.2% TritonX-100 was used. Primary antibodies (Suppl. Table 3) and phalloidin-iFluor 647 (1:500, Abcam) were diluted in blocking solution and applied overnight at 4 °C. Cells were washed with PBS and incubated for 1 h at RT with fluorescently labeled secondary antibodies (1:1,000; donkey or goat Alexa Fluor 488-, 568-, 546-, 647-labeled IgG and Alexa Fluor 488-labeled IgM, from Thermo Fisher; donkey or goat Cy3-, Cy5-labeled IgG, from Dianova) against the corresponding primary antibody species (see Suppl. Table 3). Nuclei were counterstained with DAPI (1:10,000, Sigma-Aldrich) and cells were imaged on an AxioObserver or a laser scanning microscope LSM 780 using Zen blue v. 1.1.2.0 or Zen black software v. 8.1 (Carl Zeiss), respectively. Identical illumination and acquisition conditions were applied for each experiment. Imaging and data analyses were performed blinded to the experimental conditions.

### Immunohistochemistry

Human sections were thawed for 1–2 min, rinsed in Tris-buffered saline (TBS, 50 mM Tris/HCl, pH 7.4, 150 mM NaCl) and fixed using 4% PFA for 5 min (all at RT). Prior and after all staining steps, sections were washed 2–3 times in TBS. Incubation with Phalloidin-iFluor 647 Reagent solution (1:500 in TBS) was performed for 20 min using a humid chamber. Finally, sections were stained using DAPI solution (1:10,000 in TBS) for 10 min at RT and mounted using ProLong™ Gold antifade reagent.

### Quantifications

*Cell differentiation and morphological phenotyping:* Quantification of cell populations was performed on 5–10 coverslip regions per experiment and condition using the cell counter plugin of ImageJ 1.51p [45].

*Length of myelin basic protein (MBP* +*) segments:* Confocal image stacks of GFP + hOLs plated on nanofibers were processed using ImageJ 1.51p (generation of maximum intensity projections and binary images) [45]. Maximum intensity projections of z-stacks for the PLL and MBP channels were generated and converted into binary images. Co-localized pixels were consequently identified in overlay images and retained for further analysis. The length of MBP + segments along nanofibers was determined using the particle analyzer plugin by applying the following parameters: circularity, 0–0.02; size, 10 µm – infinity. Oligodendroglial processes were counted manually. 3D reconstructions of image stacks were generated with Imaris 7.7.1 © (Bitplane).

*Length of MBP* + *segments in co-cultures:* Confocal image stacks of co-cultures were analyzed using ImageJ 1.52 k [45]. A region of interest (ROI) of 950 × 700 pixels around individual hOLs was defined and the length of MBP + neurites was manually measured in overlay images of TUBB3 and MBP channels. The ROI was transferred to the corresponding TUBB3 image and a threshold was set to identify the TUBB3 + area, followed by binarization, inversion, and particle analysis (parameters: size: 0–infinity, circularity: 0.00–1.00). Only hOLs exhibiting ensheathment events and ROIs containing > 10 K TUBB3 + pixels were included in the analysis.

*Cell size and phalloidin staining intensity *in vitro: GFP + hOLs were outlined in the center of confocal image stacks based on O4 staining using ImageJ 1.51p and MBP staining using Image J2 [44]. The cell contour was chosen as ROI and cell size was calculated based on the ROI area. The signal intensity of phalloidin staining was determined within the ROI as mean gray value after exclusion of the nucleus and background subtraction.

*Phalloidin staining intensity *in vivo: White matter striae were outlined and assessed as previously described for cell culture approaches, omitting nuclear correction. One putaminal section of human *post-mortem* tissue with ten randomly chosen WM striae per donor were assessed (total: 10 ROIs per donor). Mean gray values of all chosen white matter striae were averaged per donor prior to statistical analyses.

### Reverse transcription quantitative PCR for gene expression analysis

Total RNA was extracted from cell lysates using the RNeasy plus mini kit (Qiagen) according to manufacturer’s instructions. RNA concentration was determined by spectrometry using NanoDrop ND1000 (PeqLab). cDNA was generated using the GoScriptTM Reverse Transcription System (Promega) and reverse transcription quantitative PCR was performed on a Roche Light Cycler 480 system using the SSoFast^TM^EvaGreen® Supermix (Bio-Rad). Relative expression levels were calculated by the 2^−∆∆ct^ method and normalized to the mean of two housekeeping genes (18S rRNA, and GAPDH). Primer sequences are summarized in Suppl. Table 2.

### Flow cytometry analysis

Cells were enzymatically detached using accutase and stained using mouse IgM anti-O4-APC antibody or the corresponding isotype control (Suppl. Table 3) according to manufacturer’s instruction. Antibodies were removed by centrifugation and cells were washed twice with PBS. All centrifugation steps were performed at 300 g for 7 min. Life-dead discrimination was carried out by DAPI staining (1:10,000). Flow cytometry analysis was performed on a BD LSR FortessaTMX-20 (BD) acquiring 10,000 cells. Raw data was processed using FlowJo software. Fluorescence-activated cell sorting (FACS) for RNA isolation and protein extraction was conducted on a MoFlo Astrios EQ (Beckman Coulter).

### Western blot analyses

Cells were mechanically detached, centrifuged, and homogenized in radioimmunoprecipitation assay (RIPA) buffer (50 mM Tris/HCl, pH 8.0, 150 mM NaCl, 2 mM EDTA, 1% NP-40, 0.1% SDS, and 0.5% sodium deoxycholate). Fresh frozen *post-mortem* tissue was homogenized in RIPA buffer as well. Protein concentration was determined using the Pierce® BCA protein assay kit (Thermo Scientific) according to manufacturer’s instructions. Total protein (6 µg for unsorted hOLs, 12 µg for sorted hOLs and 20 µg for *post-mortem* tissue samples) was separated on NuPAGE^TM ^4–12% Bis–Tris gels (Invitrogen) and blotted onto an EMD Millipore Immobilon™^−^FL PVDF Transfer Membrane (Merck Millipore). Membranes were fixed with 4% PFA in TBS (30 min) and blocked with TBS / 1% BSA / 0.1% Tween for 1 h at RT followed by the incubation with respective primary antibodies (Suppl. Table 3) overnight at 4 °C. Membranes were washed with TBS / 0.1% Tween and fluorescently labeled secondary antibodies (1:1,000, donkey or goat Alexa Fluor 488-, Alexa Fluor 647-labeled IgG, from Thermo Fisher; 1:10,000, donkey or goat 680RD-, 800CW-labeled IgG, from LI-COR) against the corresponding primary antibody species (see Suppl. Table 3) were applied for 1 h at RT. Fluorescent signals were captured with the Fusion FX7 (PeqLab), the LI-COR Odyssey M (LI-COR), or the iBright 1500 (Invitrogen) detection system and densitometric analysis was performed using the Bio1D (Vilber Lourmat), the Image Studio 5.5 (LI-COR), or the Empiria Studio 3.3 (LI-COR) software. Relative expression levels were calculated by normalizing arbitrary units (AU) to GAPDH signal.

### Generation of lentiviral particles

Third-generation lentiviral particles coding for human aSyn (NM 000345) and/or an internal ribosomal entry site (ires) followed by the coding sequence of GFP under the control of the EF1α-promoter were produced as previously described [10]. Following transfection and virus particle assembly in HEK293T cells, virus-containing supernatant was harvested after 48 h and filtered through a 0.45 µm PVDF membrane. Lentiviral particles were concentrated by ultracentrifugation at 28,000 rpm for 2 h at 4 °C.

### Statistical analyses

Data were processed and visualized using OriginPro, Version 2021 and GraphPad Prism® 6.07, respectively. If not stated differently, data were obtained from four hOL lines derived from two individuals. Bar graphs depict the mean + standard deviation (SD) and box plots represent the median and the 25./75. percentile. Corresponding whiskers indicate the 5./95. percentile. The Shapiro–Wilk test was used to assess the normal distribution of the parameters for small sample sizes (*n* < 20). Additionally, the F-test was applied to test for equality of variances. Statistical significance was determined by one-way analysis of variance (ANOVA), two-way ANOVA, Kruskal–Wallis test, two-tailed student’s t-test, Mann–Whitney *U*-test, or one sample *t*-test. The respective tests are indicated in the figure legends. *P* values < 0.05 were considered statistically significant (*p < 0.05, **p < 0.01, ***p < 0.001).

## Results

### Deterministic forward programming of hiPSCs into hOLs by forced expression of SOX10, OLIG2, and NKX6.2 (SON)

**Fig. 1:**
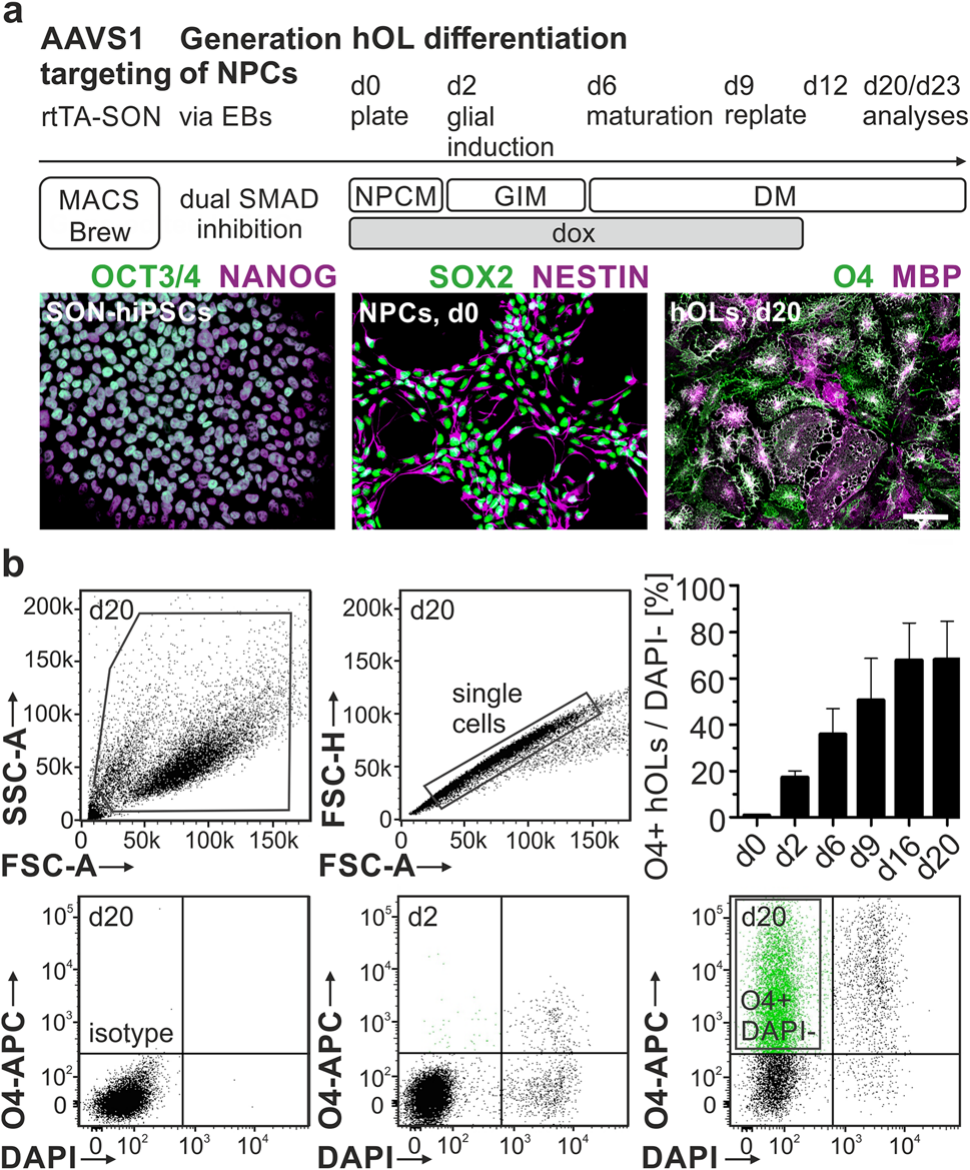
Forward programming of human induced pluripotent stem cells (hiPSCs) into human oligodendrocytes (hOLs). **a** Differentiation paradigm of hOLs. Gene-edited hiPSCs express the pluripotent stem cell markers NANOG and OCT3/4. Small molecule-mediated dual SMAD inhibition and culture as free-floating embryoid bodies (EB) induces the differentiation of hiPSCs into neural precursor cells (NPCs) expressing the neuroectodermal markers SOX2 and NESTIN. hOLs express the oligodendroglial lineage markers O4 and myelin basic protein (MBP). **b** Flow cytometry analysis of the O4 epitope on hOLs upon SON induction. Data are presented as mean + standard deviation (SD). Mean values for four independent differentiation experiments were included for each hOL line (*n* = 4). Scale bars: 50 µm. *APC* allophycocyanin, *AAVS1* Adeno-Associated Virus Integration Site 1, *d* day, *DAPI* 4′,6-Diamidin-2-phenylindol, *DM* differentiation medium, *dox* doxycycline, *FSC-A / -H* forward scatter area / height, *GIM* glial induction medium, *NPCM* NPC medium, *rtTA* reverse tetracycline transactivator, *SON* SOX10, OLIG2, NKX6.2, *SSC-A* side scatter area

hiPSCs represent an important source for the generation of myelinating oligodendrocytes. Besides the sole application of extrinsic growth factors and morphogens, distinct sets of cell fate-determining transcription factors are able to promote oligodendroglial lineage specification in hiPSCs and neural precursor cells [9, 14, 34]. Previously, the combined ectopic expression of SON resulted in a robust generation of oligodendrocytes from hiPSC-derived NPCs [9]. Stable and inducible SON-hiPSC lines were successfully generated via zink-finger assisted and site-directed integration of the expression cassette into the AAVS1 genomic safe harbor locus [15]. Here, we created SON-hiPSC-lines of two healthy individuals (C1, C2) and subsequently expanded two hiPSC-clones per individual (C1A/B, C2A/B). The SON knock-in lines displayed typical hiPSC morphology with a constant expression of the pluripotency markers OCT3/4 and NANOG (Fig. 1a). Upon small-molecule mediated inhibition of the bone morphogenic protein (BMP) and transforming growth factor beta (TGFb) pathways (dual SMAD inhibition) [9, 41], we obtained NPCs in high purity confirmed by the expression of the neuroectodermal stem cell markers SOX2 and NESTIN (Fig. 1a).

**Fig. 2:**
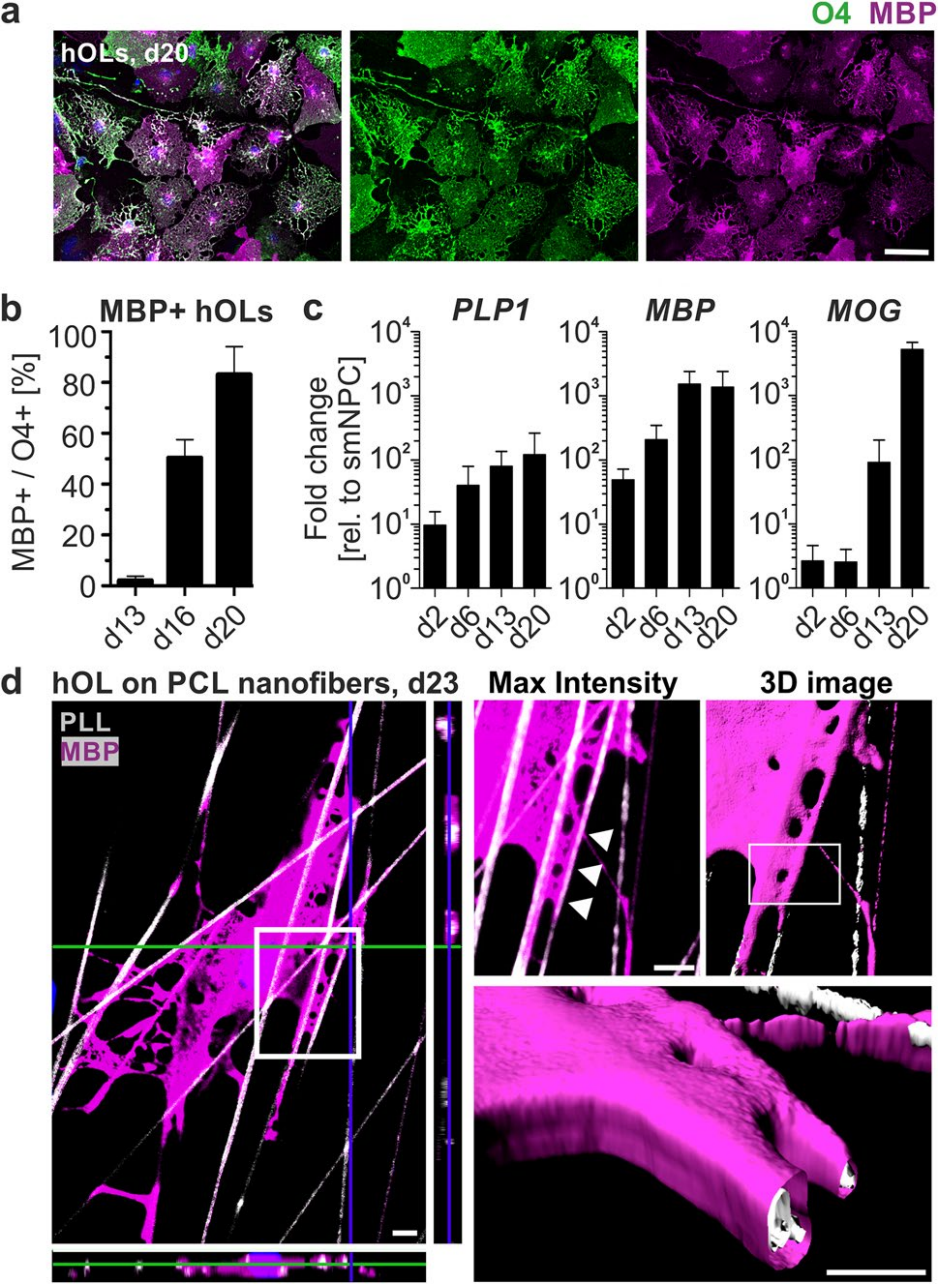
Phenotypical and functional characterization of hOLs. **a** Representative images and **b** Quantification of O4 + hOLs co-expressing MBP. **c** Temporal gene expression pattern of the myelin genes *PLP1*, *MBP*, and myelin oligodendrocyte glycoprotein (*MOG*). **d** Representative image of MBP + hOL cultured on aligned polycaprolactone (PCL) nanofibers coated with Cy5-labeled poly-L-lysin (PLL) and three-dimensional (3D) image reconstruction. MBP + myelin segments are visualized along nanofibers (arrowheads). Data are presented as mean + SD. Mean values for three independent differentiation experiments were included for each hOL line (*n* = 4). Scale bars: 50 µm (**a**), 5 µm (**d**)

For oligodendroglial lineage specification, NPCs were treated with dox for twelve days followed by a two-step differentiation protocol (Fig. 1a). Using this approach, NPCs efficiently differentiated into oligodendroglial lineage cells expressing the oligodendrocyte marker O4 followed by MBP indicative for hOLs with myelinogenic potential (Fig. 1a). Characterizing the dynamics of oligodendroglial differentiation, we performed flow cytometry analysis for the O4 epitope (Fig. 1b). The different NPC lines demonstrated a progressive conversion to the oligodendroglial lineage, starting from 17.2 ± 2.7% O4 + cells at day 2 to 68.1 ± 16.6% O4 + cells by day 20. Next, we performed co-labeling of O4 and MBP to further evaluate the oligodendroglial phenotype in vitro (Fig. 2a). While only a small proportion of O4 + cells co-expressed MBP on day 13 (2 ± 1.8%), the level of MBP expression of the O4 + cells steadily increased until day 20, when up to 83% of O4 + cells co-expressed MBP (Fig. 2b). The concomitant transcriptional upregulation of *MBP*, proteolipid protein 1 (*PLP1)*, and myelin oligodendrocyte glycoprotein (*MOG*) suggested a strong promyelinogenic potential of hOLs (Fig. 2c), which was further assessed by culturing hOLs on PCL nanofibers modelling a three-dimensional (3D) axonal-like scaffold structure. Indeed, hOLs extended multiple MBP-positive processes from the cell body toward nanofibers with multiple segments of ensheathment visualized by a 3D reconstruction (Fig. 2d).

### aSyn does not induce cellular toxicity or a differentiation deficit in hOLs, but induces an intraoligodendrogial redistribution of TPPP/p25α

**Fig. 3:**
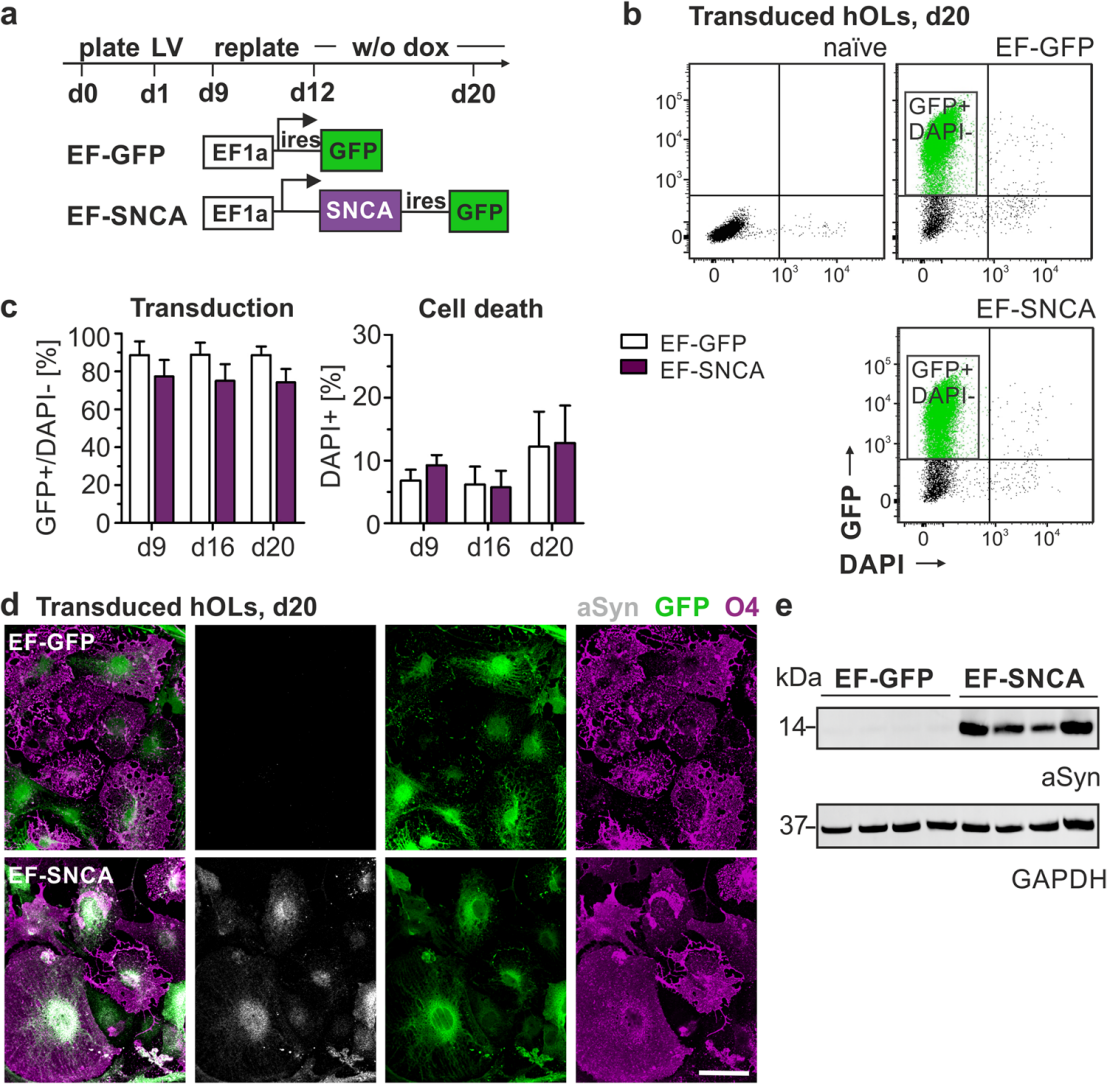
Effects of lentiviral *SNCA* transduction on aSyn expression in hOLs. **a** Experimental design of lentivirus-mediated overexpression of human aSyn (EF-SNCA) or green fluorescent protein (EF-GFP) in hOLs driven by the eukaryotic elongation factor 1 alpha (EF1a) promoter. **b–c** Flow cytometry analysis of hOLs confirmed a high transduction efficiency for both constructs. Cytotoxicity was quantified using DAPI, which is indicative of cell death. Data are presented as mean + SD. Mean values of three independent transduction experiments were included for each individual hOL line (*n* = 4). **d** Representative images of intracellular aSyn, GFP, and O4 expression upon lentiviral transduction and (**e**) complementary Western blot analysis for aSyn (*n* = 4). GAPDH (glyceraldehyde-3-phosphate dehydrogenase) served as loading control. Scale bar: 50 µm. *ires* internal ribosomal entry site

**Fig. 4:**
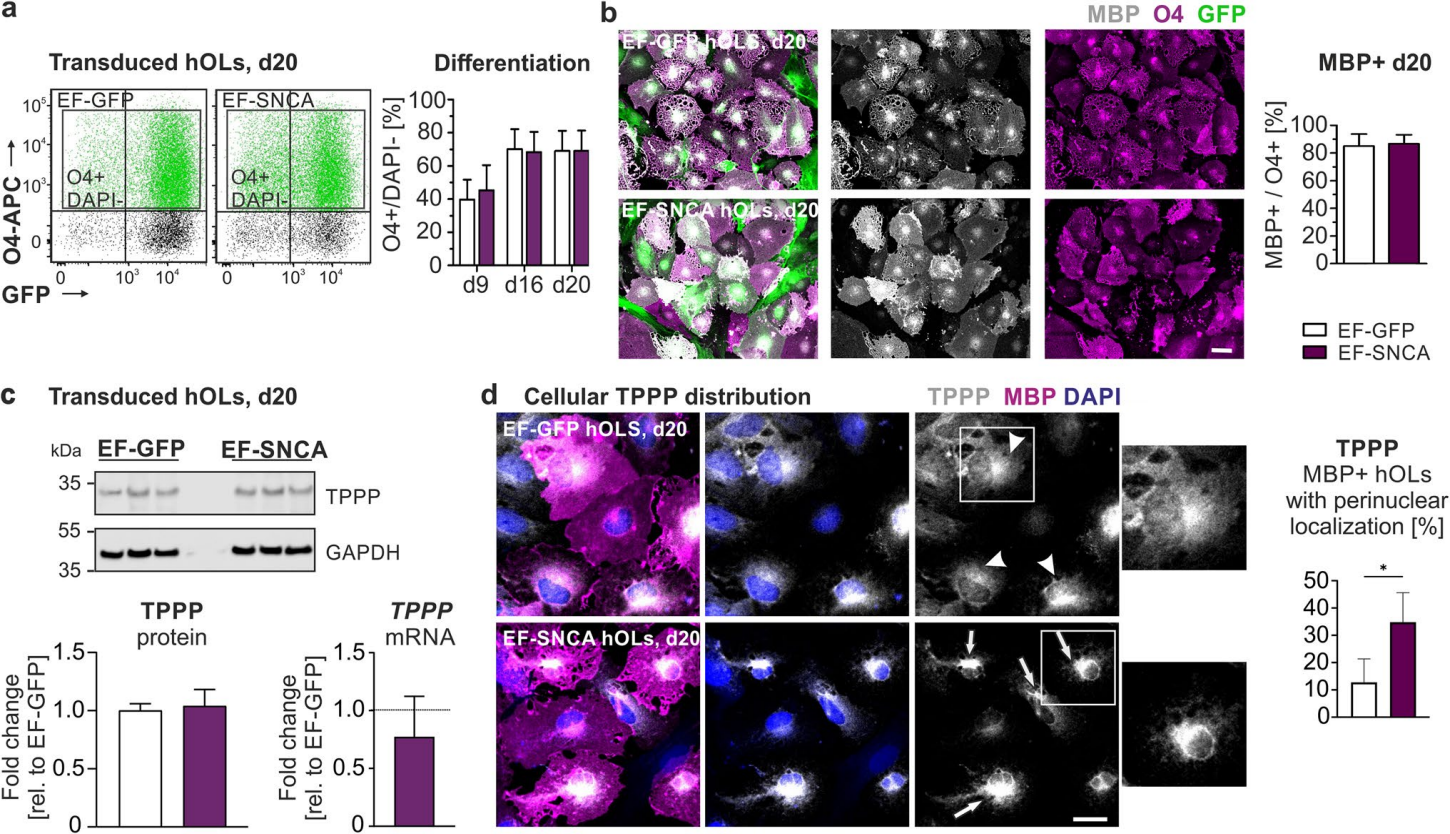
Effect of ectopic *SNCA* overexpression on hOL differentiation and TPPP. **a** Quantification of O4 + cells during differentiation based on flow cytometry analysis. Two-way ANOVA followed by Bonferroni post-hoc test. F (groups) = 0.063, *p* = 0.805. Mean values of three independent transduction experiments were included for each hOL line (*n* = 4). **b** Representative images and quantification of O4 + hOLs co-expressing MBP. Scale bar: 50 µm. Student’s *t*-test. *p* (MBP) = 0.614, *n* = 4. **c** Western blot and gene expression analyses for TPPP in transduced, sorted O4 + /GFP + hOLs at day 20 of differentiation. For Western blot, GAPDH served as loading control, Student’s *t*-test, *p* (TPPP protein) = 0.682, *n* = 3; For gene expression analysis, one sample *t*-test, *p* (TPPP mRNA) = 0.300, *n* = 4. **d** Representative images of TPPP distribution in MBP + cells differentiated for 20 days. MBP + hOLs exhibiting diffuse cytoplasmic patterns and perinuclear localization (with ring-like structures and/or intense perinuclear clusters) are highlighted by arrowheads and arrows, respectively. The percentage of MBP + hOLs with perinuclear localization is shown. Scale bar: 20 µm. Mann Whitney *U* test, *p* = 0.0286, *n* = 4 (38–78 cells of each hOL line). Bar graphs represent the mean + SD. p* < 0.05

Intraoligodendroglial accumulation of aSyn is the cytopathological hallmark in MSA. The origin of this accumulation, however, remains a current matter of debate. Previous studies using rat primary oligodendrocytes cultures and hiPSC-derived oligodendrocytes have demonstrated a transient expression of aSyn during oligodendrocyte differentiation [2, 7, 42]. To further investigate endogenous aSyn expression in SON-induced hOLs, we performed immunocytochemical and transcriptional analyses during differentiation. Notably, although sparsely expressed, aSyn was detected in O4 + hOLs with a predominant perinuclear localization (Suppl. Figure 1a, arrowheads). Correspondingly, a transient upregulation of *SNCA* mRNA was detected, reaching its maximum at day 13 (Suppl. Figure 1b). The presence of endogenous aSyn, as well as its transient upregulation, was further supported by Western blot analysis of hOLs differentiated for 16 and 20 days, although only at very low levels (Suppl. Figure 1c). To investigate the effect of high levels of intraoligodendrocytic aSyn on human oligodendrocyte differentiation and function, we made use of a previously described lentiviral bicistronic vector system expressing human aSyn-ires-GFP or ires-GFP under the control of the elongation factor 1a (EF1a) promotor [10, 11] (Fig. 3a, referred as to EF-SNCA and EF-GFP, respectively). hOLs demonstrated a high proportion of GFP + cells following transduction with the corresponding lentiviral vector as confirmed by flow cytometry (Fig. 3b/c). DAPI staining was implemented to exclude toxic effects of aSyn expression or the viral construct on cell survival. Both, immunocytochemistry and Western blotting confirmed a robust expression of aSyn in EF-SNCA-transduced cells whereas aSyn was barely present in EF-GFP-transduced hOLs (Fig. 3d/e). Again, aSyn expression was primarily detected in the cytoplasm of EF-SNCA-transduced hOLs with a predominant enrichment in the perinuclear region (Suppl. Figure 2) matching the observed intracellular distribution pattern of endogenous aSyn. To explore whether aSyn interferes with the in vitro differentiation of hOLs, we examined the temporal pattern of oligodendrocyte lineage commitment in EF-SNCA- and EF-GFP-transduced cultures by determining the proportion of O4 + cells using flow cytometry (Fig. 4a). However, there was no effect on the number of O4 + hOLs and their capacity to differentiate into MBP expressing hOLs (Fig. 4b).

TPPP is highly enriched in myelinating oligodendrocytes [24, 49]. While under physiological conditions TPPP interacts with tubulin and MBP to promote myelination, it is present within GCIs and assumed to promote aSyn aggregation in MSA [22, 23, 53]. Given its physiological and pathological relevance, we analyzed TPPP in response to EF-SNCA transduction. No significant differences were observed in TPPP mRNA and protein levels, following aSyn overexpression in hOLs (Fig. 4c). Similarly, in *post-mortem* putamen of MSA patients, TPPP protein levels were not significantly altered (Suppl. Figure 3). We next examined the intraoligodendroglial distribution of TPPP in MBP + hOLs using immunocytochemistry. TPPP exhibited several distinct patterns, including (1) diffuse cytoplasmic distribution, (2) perinuclear ring-like structures, and/or (3) perinuclear intense clusters (Fig. 4d). In EF-GFP-transduced cells, TPPP predominantly displayed a diffuse cytoplasmic pattern (Fig. 4d, arrowheads). In contrast, EF-SNCA–transduced cells showed a significantly increased perinuclear accumulation, including perinuclear ring-like and/or intense cluster patterns (Fig. 4d, arrows). Taken together, these results indicate that EF-SNCA transduction induces an intraoligodendroglial redistribution of TPPP toward perinuclear regions, despite unchanged overall TPPP expression.

### aSyn alters process outgrowth and shape of hOLs

**Fig. 5:**
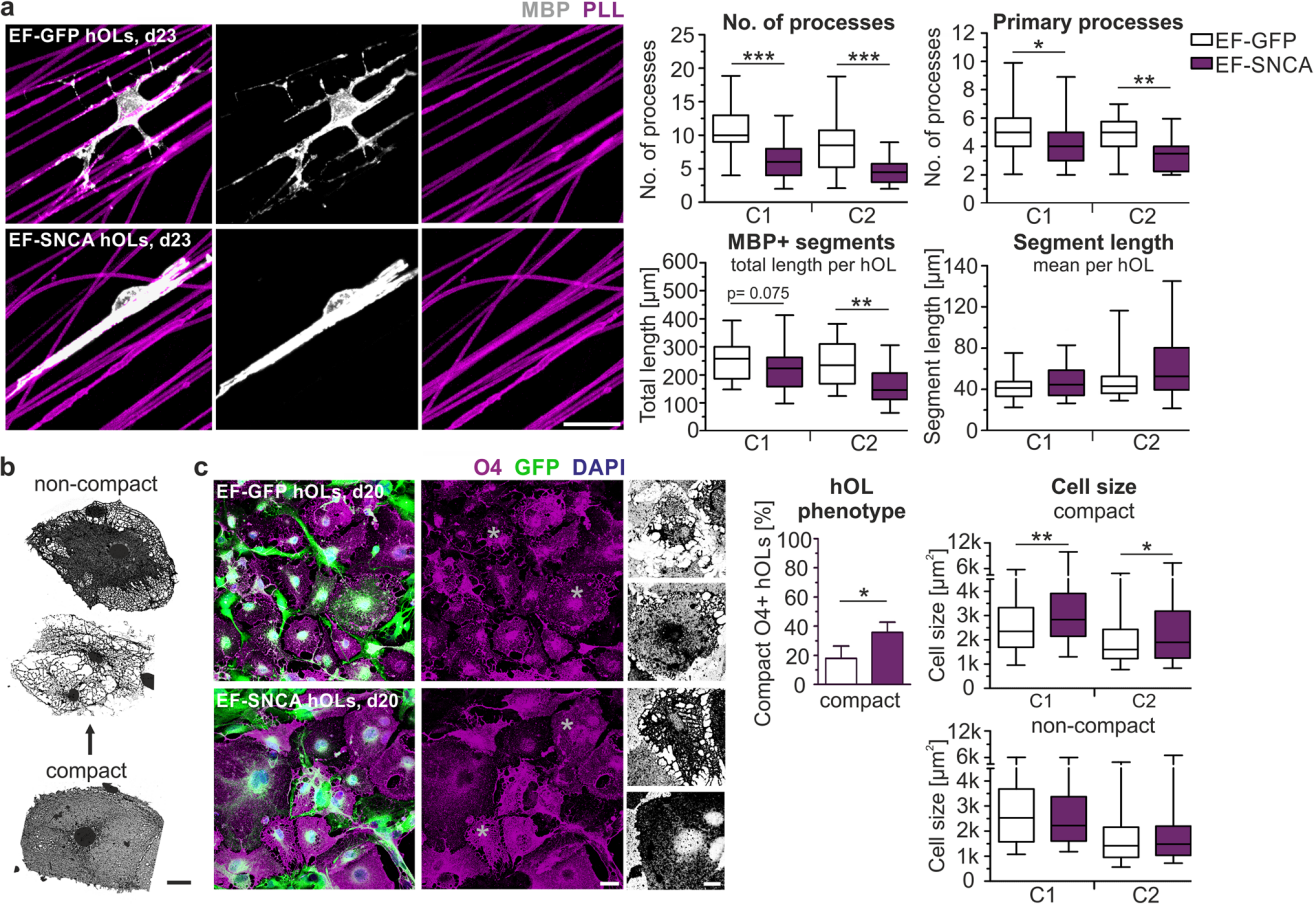
aSyn overexpression affects oligodendrocyte morphology and the potential to ensheath axon-mimicking structures. **a** Representative images of transduced MBP + hOLs cultured on aligned PCL nanofibers coated with Cy5-labeled PLL (day 23). Depicted is the quantification of the number of total processes as well as primary processes, the total length of MBP + segments per hOL, and the average length of individual segments. Student’s *t*-test, *p* (no. of processes) C1 / C2 < 0.001; *p* (primary processes) C1 = 0.047 / C2 = 0.001; *p* (total length MBP + segments along nanofibers) C1 = 0.075 / C2 = 0.003; *p* (segment length) C1 = 0.151 / C2 = 0.092; *n* = 40 (C1), 20 (C2). **b** O4 + /GFP + hOLs are distinguished into two morphological subtypes: compact hOLs without defined processes and non-compact hOLs with or without membranes. **c** Morphological phenotyping of transduced hOLs at day 20. Asterisks mark the area of magnification boxes (right panel). Quantification of compact O4 + hOLs and cell size of compact and non-compact hOLs based on O4 immunoreactivity. Student’s *t*-test, *p* (% compact) = 0.018, *n* = 4, *p* (compact, size) C1 = 0.006 / C2 = 0.024, and *p* (non-compact, size) C1 = 0.602 / C2 = 0.816, *n* = 120. Bar graphs represent the mean + SD. Box plots show the 25. / 75. percentile; whiskers of the box plots indicate the 5. / 95. percentile. Scale bars: 20 µm (**a**/**b**) and 50 µm (**c**). p* < 0.05, p** < 0.01, p*** < 0.001

During maturation, oligodendrocytes undergo profound and dynamic structural changes in shape and size before forming myelin sheaths [8, 28, 66]. To explore potential effects of aSyn overexpression on these processes, we cultured transduced hOLs on aligned PCL nanofibers and analyzed the formation of MBP + segments at a single cell level. Our findings revealed a 39% (C1) and 47% (C2) decrease in the number of processes, a 16% (C1) and 28% (C2) reduction in the number of primary processes, and a 13% (C1) and 32% (C2) decrease in the total length of MBP + segments per hOL upon ectopic aSyn expression (Fig. 5a). The length of individual MBP + segments was, however, unaltered suggesting that the overall reduction in MBP + segment length is more likely due to a decreased process outgrowth rather than impaired MBP + segment formation and elongation along nanofibers. These observations prompted us to investigate whether EF-SNCA-transduced hOLs undergo specific structural changes during differentiation. Based on previously established criteria for primary rodent OPCs [66], we subdivided hOLs into two cellular phenotypes: (1) compact: oligodendrocytes without defined processes but with membranous structures and (2) non-compact: branched oligodendrocytes without membrane sheets or with a partial formation of myelin membranes (Fig. 5b). Both hOL subtypes were reliably identified using the oligodendroglial lineage markers O4 and MBP (Fig. 5b, and Suppl. Figure 4). To characterize the effect of EF-SNCA transduction on morphological changes, we analyzed O4 + /GFP + hOLs after 20 days differentiation. O4 was chosen as oligodendroglial lineage marker because it also labels less mature cells not yet expressing MBP (Fig. 2b), thereby providing a more comprehensive structural overview of distinct hOL subtypes. The classification of O4 + /GFP + hOLs into both cellular phenotypes revealed an almost doubled proportion of compact oligodendrocytes in EF-SNCA-transduced cultures (Fig. 5c). Moreover, the identified compact hOLs showed a larger cell size upon ectopic aSyn overexpression (increase of 40% and 26% for C1 and C2, respectively). Taken together, these findings indicate an aSyn-mediated effect on the shape of oligodendrocytes and the process outgrowth in vitro.

### aSyn affects the number of axon-ensheathing hOLs in a human triple-culture of cortical neurons, astrocytes, and hOLs

**Fig. 6:**
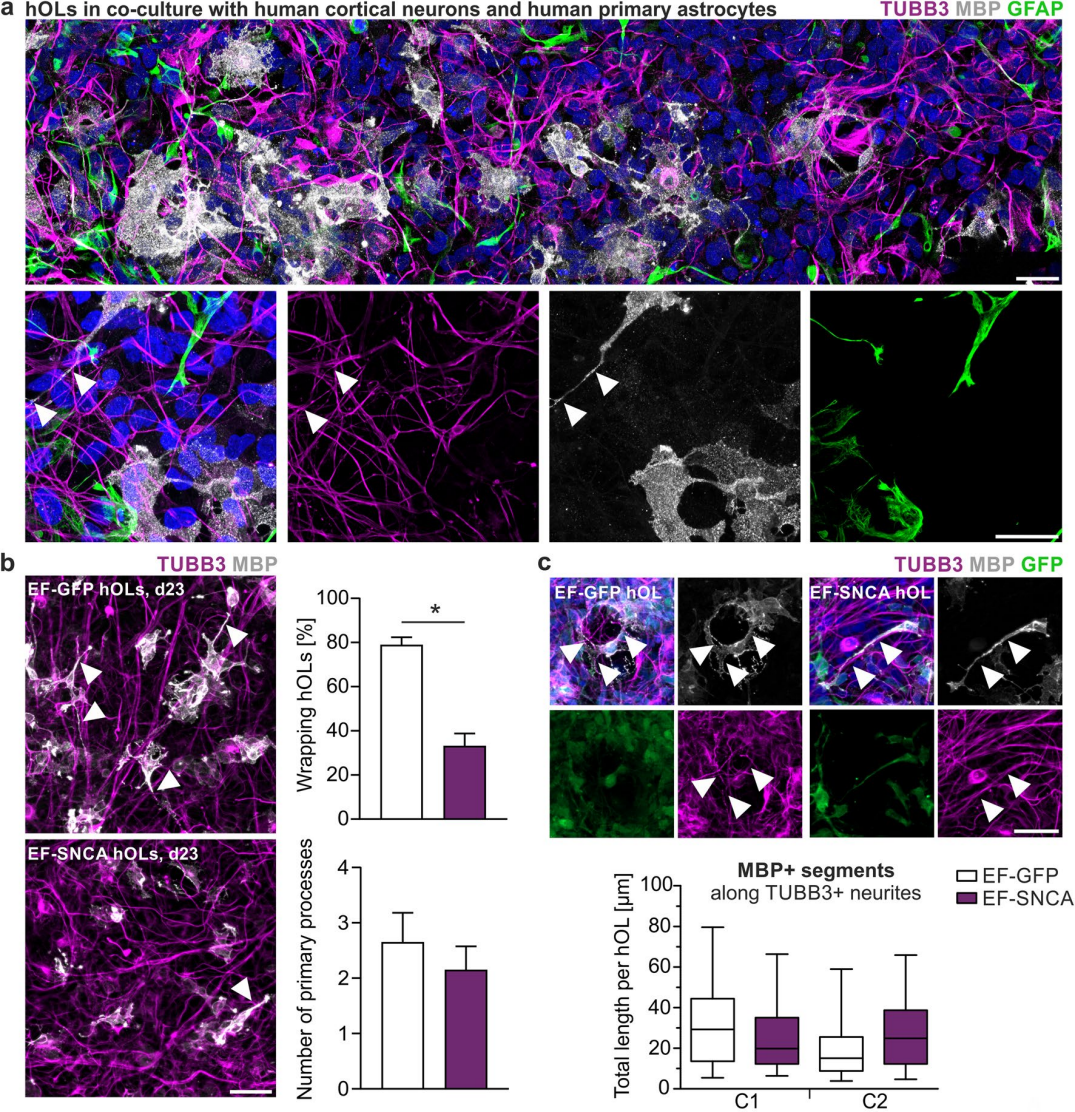
aSyn overexpression affects the number of axon-ensheathing hOLs. **a** Representative images of hOLs (d23) in co-culture with human cortical neurons and human primary astrocytes, visualized by MBP, beta-III-tubulin (TUBB3), and GFAP, respectively. Cells were co-cultivated for 14 days. White arrowheads indicate exemplary events of axon-ensheathment. **b**–**c** Representative images of transduced MBP + hOLs in co-cultures. White arrowheads indicate exemplary events of axon-ensheathment. **b** Depicted is the reduction of neurite-ensheathing EF-SNCA hOLs (%) as well as the number of extended primary processes per hOL. Mann–Whitney *U*-test, *p* (wrapping hOLs) = 0.030, *n* = 4. Student’s *t*-test, *p* (primary processes) = 0.192, *n* = 4. **c** Total length of MBP + segments along TUBB3-positive neurites per hOL. Only hOLs with wrapping events were analyzed. Student’s *t*-test, *p* (total length MBP + segment) C1 = 0.176 / C2 = 0.070, *n* = 48. Bar graphs represent the mean + SD and box plots the 25. / 75. percentile. Whiskers of the box plots indicate the 5. / 95. percentile. Scale bars: 25 µm (**a**) and 50 µm (b-c). p* < 0.05

The cultivation of hOLs on inert nanofibers enables the exploration of neuron-independent intrinsic mechanisms driving myelin formation. However, myelination is a multifaceted process influenced by numerous cellular, molecular, and environmental factors, with neuronal signals such as electrical activity and growth factors playing pivotal roles in promoting oligodendrocyte differentiation and myelin formation [35, 38, 47]. To investigate the impact of aSyn on process outgrowth and myelin ensheathment within a more physiologically relevant environment, a human triple-culture was developed comprising hOLs, hiPSC-derived cortical neurons, and human primary astrocytes. Immunostaining for the cell type-specific antigens MBP, beta-III-tubulin (TUBB3) as neuronal and glial fibrillary acidic protein (GFAP) as astrocytic marker, after two weeks of cultivation confirmed the survival of all three cell types (Fig. 6a) and demonstrated events of axon ensheathment identified as MBP + segments along TUBB3 + neurites (Fig. 6, white arrowheads). Following EF-GFP and EF-SNCA transduction, we evaluated the percentage of hOLs exhibiting ensheathment events and quantified the number of primary processes per hOL within the triple-culture. Notably, aSyn overexpression led to a 58% reduction in the proportion of ensheathing hOLs suggesting a significantly impaired myelinogenic potential. Additionally, while a slight 14–23% reduction in the average number of primary processes extended by EF-SNCA-transduced hOLs was observed across all lines, this decrease was not statistically significant (Fig. 6b). We then analyzed the length of MBP + segments along TUBB3 + neurites at a single-cell level, focusing exclusively on hOLs exhibiting ensheathment events. hOLs without detectable MBP + segments were excluded from this analysis. Interestingly, the length of ensheathing MBP + segments was not diminished following aSyn overexpression; instead, in two hOL lines derived from the identical donor a slight increase was observed (Fig. 6c, C2). In summary, the findings demonstrate a detrimental effect of aSyn overexpression on the overall capacity of hOLs to ensheath neurites within a more physiological environment, thereby corroborating observations from the hOL cultures on inert nanofibers.

### aSyn favors actin assembly in hOLs in vitro and in *post-mortem* MSA tissue

**Fig. 7:**
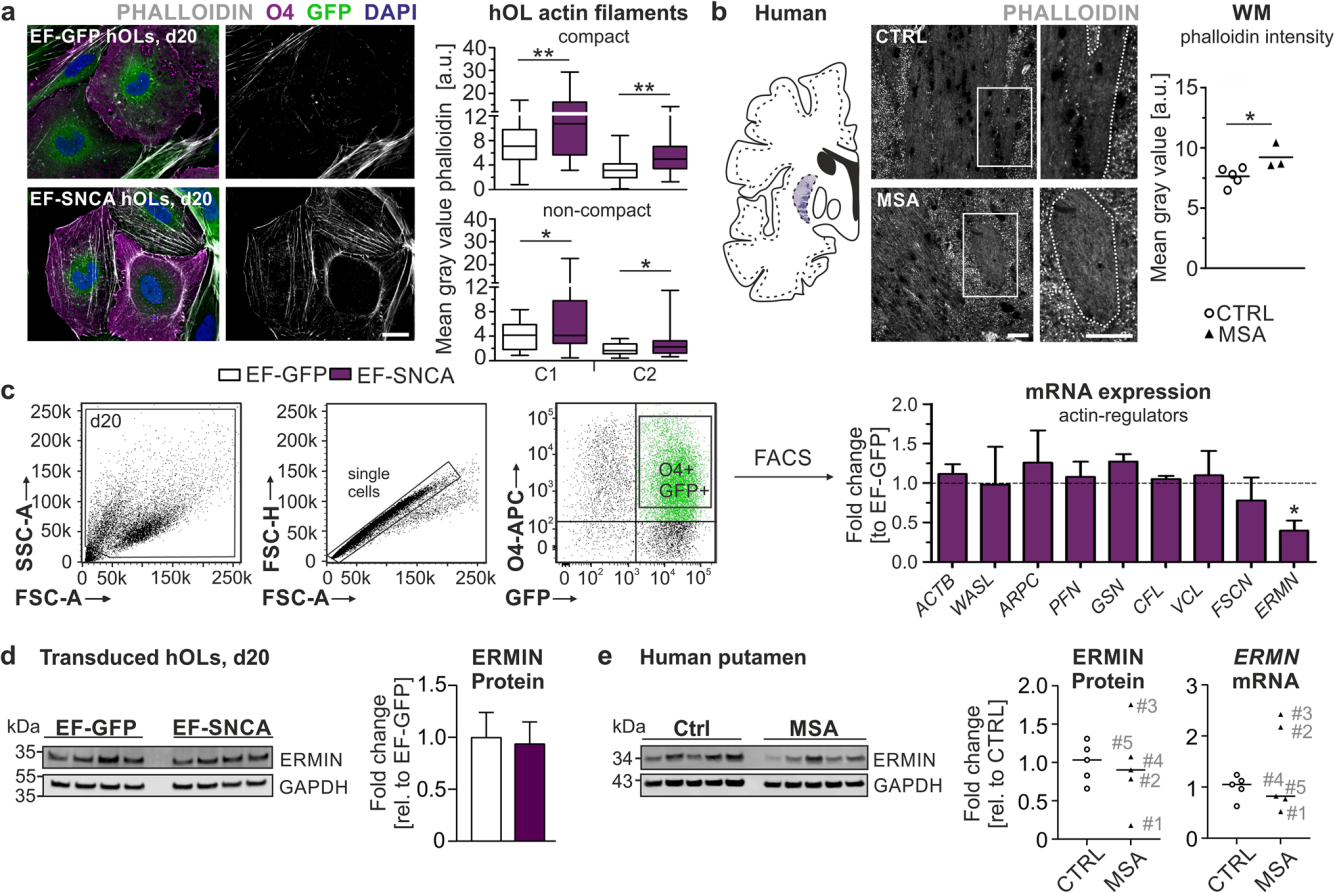
Effects of aSyn on the oligodendroglial actin cytoskeleton. **a** Transduced hOLs stained for O4 and actin filaments using fluorophore-coupled phalloidin (day 20). Quantification of actin filament levels at a single cell level based on phalloidin signal intensity. Box plots show the 25. / 75. percentile; whiskers of the box plots indicate the 5. / 95. percentile. Student’s *t*-test, *p* (phalloidin intensity—compact): C1 = 0.002 / C2 = 0.004, *n* = 40; *p* (phalloidin intensity—non-compact): C1 = 0.014 / C2 = 0.018, *n* = 40. **b** Coronal brain sections of *post-mortem* putamen of patients with MSA (*n* = 3) and controls (CTRL, *n* = 5) were stained using fluorophore-coupled phalloidin. Phalloidin signal intensity was quantified in putaminal white matter (WM, dashed lines) tracts. Scatter plots depict the individual means per patient. Student’s t-test, *p* (actin, human) = 0.046. **c** Gene expression levels of actin regulators in transduced, sorted O4 +/GFP + hOLs at day 20. Bar graphs represent the mean + SD. One sample t-test with Bonferroni correction for multiple testing (*p* < 0.05/k; *k* = 9). *P* values < 0.0056 were considered statistically significant. *p* (beta-actin, ACTB) = 0.1659, *p* (neural wiskott-aldrich syndrome protein, WASL) = 0.9478, *p* (actin-related protein 2/3 complex, ARPC) = 0.2946, *p* (profilin, PFN) = 0.4656, *p* (gelsolin, GSN) = 0.0102, *p* (cofilin, CFL) = 0.0825, *p* (vinculin, VCL) = 0.5703, *p* (fascin, FSCN) = 0.2282, *p* (ERMIN, ERMN) = 0.0027, *n* = 4. **d** Western blot analysis for ERMIN in transduced, sorted O4 +/GFP + hOLs at day 20. GAPDH served as loading control. Bar graphs represent the mean + SD. Student’s *t*-test, *p* (ERMIN in hOLs) = 0.718, *n* = 4. **e** Western blot and mRNA analyses for ERMIN in putaminal *post-mortem* tissue of MSA patients and controls. Mann–Whitney *U* test, *p* (*ERMN* mRNA) > 0.9999, *p* (ERMIN protein) = 0.8413, *n* = 5/group, with #1–5 indicating individual MSA patients. Scatter plots depict individual values of individuals and means per group. Scale bars: 50 µm. p* < 0.05, p** < 0.01; **c** p* < 0.005

The cellular shape of eukaryotic cells is primarily determined by the dynamic organization of the actin cytoskeleton [56]. Given that increased aSyn levels alter hOL morphology, we examined the actin network by applying fluorophore-coupled phalloidin. Analysis of phalloidin intensity in MBP + cells revealed a significant increase in phalloidin signal intensity in EF-SNCA-transduced cells (Suppl. Figure 5). In a more detailed analysis, O4 was used as a marker of oligodendroglial lineage cells, which also labels less mature hOLs. O4 + /GFP + hOLs were then grouped into either the compact or the non-compact phenotype, and phalloidin signal intensity was quantified at a single-cell level. The analysis of phalloidin intensity revealed an increase in actin filaments present in EF-SNCA-transduced hOLs (Fig. 7a) suggesting that aSyn either directly or indirectly affects the balance of actin in favor of filament assembly and stabilization. To confirm these in vitro findings, we quantified the phalloidin signal intensities of actin filaments in *post-mortem* putamen, a brain region primarily affected in MSA (Fig. 7b). Consistently, the signal intensity of phalloidin in the putaminal white matter of MSA patients was significantly increased when compared to age-matched healthy donors. Taken together, we discovered an increase in the levels of actin filaments in aSyn overexpressing hOLs corresponding to white matter changes in the basal ganglia of MSA patients.

### Inhibition of the rho-associated protein kinase (ROCK) rescues the aSyn phenotype in human oligodendrocytes

**Fig. 8:**
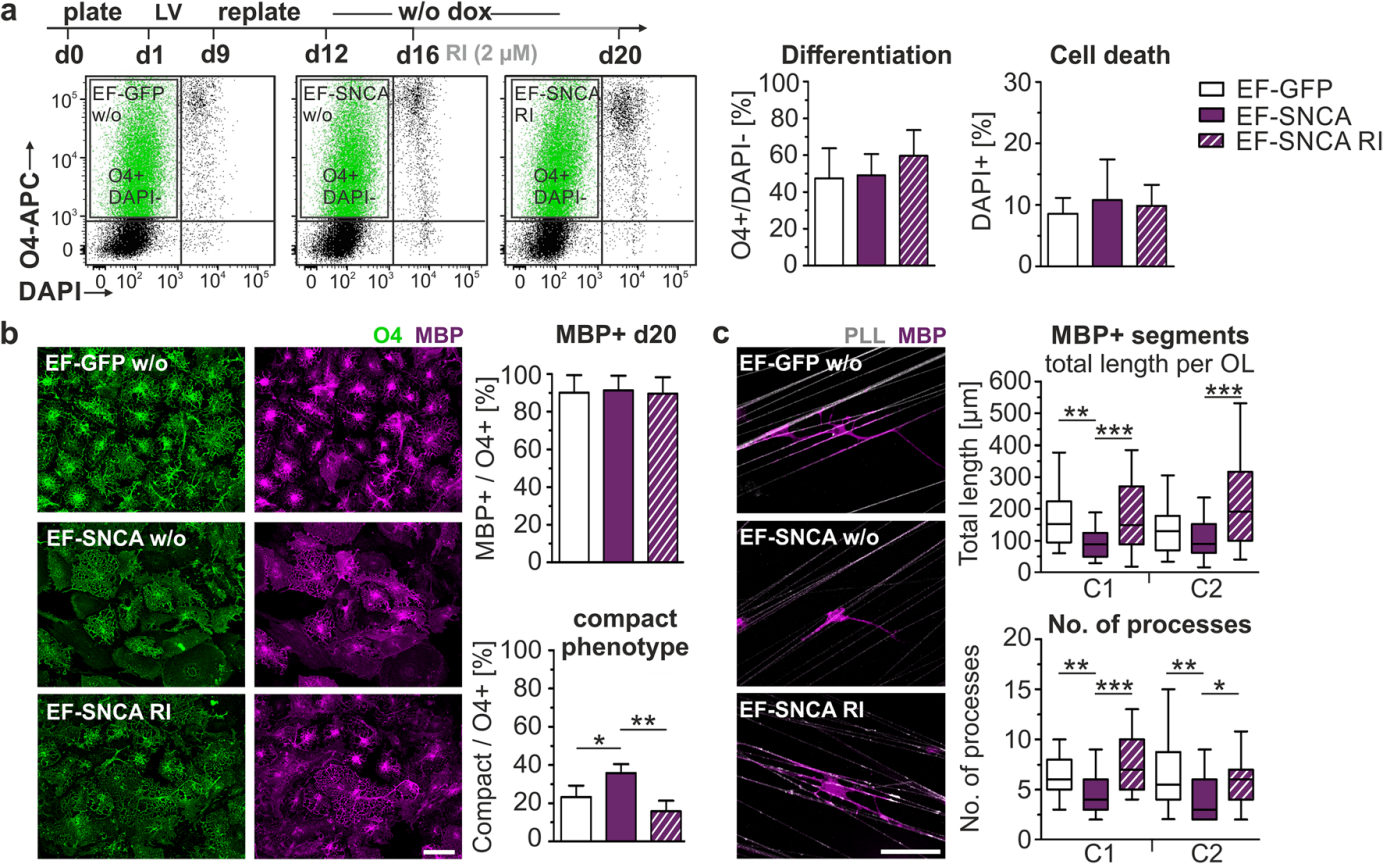
Inhibition of the Rho-associated protein kinase (ROCK) rescues the phenotypes of EF-SNCA-transduced hOLs. **a** Experimental paradigm for the treatment of transduced hOL cultures with Y–27632 (ROCK inhibitor, RI). Quantification of O4 + cells at day 20. Cytotoxicity was evaluated using DAPI, which is indicative of cell death. One-way ANOVA with Bonferroni’s multiple-comparisons test, F (O4) = 0.87, *p* = 0.451; F (DAPI) = 0.24, *p* = 0.791. Mean values of two independent differentiations were included for each individual hOL line (*n* = 4). **b** Representative images of transduced hOLs after treatment with RI. Quantification of morphological subtypes and O4 + hOLs co-expressing MBP. Kruskal–Wallis test with Dunn’s multiple-comparisons test, *p* (MBP) = 0.828, *n* = 4. One-way ANOVA with Bonferroni’s multiple-comparisons test, F (compact) = 13.803, *p* = 0.002, *n* = 4. **c** Representative images of transduced MBP + hOLs cultured on aligned PCL nanofibers coated with Cy5-labeled PLL after 4 days of treatment (day 20). Box plots depict the quantification of the total length of MBP + segments and the number of processes per hOL. One-way ANOVA with Bonferroni’s multiple-comparisons test, F (total length myelin-like segments) C1 = 9.139, *p* < 0.001 / C2 = 12.68, *p* < 0.001; F (no. of processes) C1 = 13.12, *p* < 0.001 / C2 = 7.29, *p* = 0.001, *n* = 40. Bar graphs represent the mean + SD and box plots the 25. / 75. percentile. Whiskers of the box plots indicate the 5. / 95. percentile. Scale bars: 50 µm. p* < 0.05, p** < 0.01, p*** < 0.001

Adaptive cytoskeletal changes are mediated by various actin regulators [62]. Aiming to decipher potential effects of aSyn on the regulatory network, we purified O4 + /GFP + hOLs by FACS and evaluated gene expression levels of actin regulators involved in nucleation and branching (*WASL*, *ARPC*, *PFN*), capping and severing (*GSN*, *CFL*), crosslinking (*FSCN*), membrane anchoring (*VCL*), and filament stabilization (*ERMN,* Fig. 7c). aSyn expressing hOLs exhibited a profound reduction in ERMIN transcripts (*ERMN),* a protein involved in cytoskeletal remodeling and myelin maintenance by counteracting RhoA signaling [6, 59]. However, Western blot analysis showed no significant differences in ERMIN protein levels in transduced hOLs (Fig. 7d). Furthermore, we did not observe significant differences in ERMIN mRNA or protein levels between age-matched MSA and control groups (Fig. 7e). Similarly, when comparing individual MSA cases, no profound reduction in ERMIN mRNA relative to protein was apparent. The present finding in EF-SNCA transduced hOLs, showing downregulation of ERMIN gene expression without changes at the protein level, did not clearly support ERMIN as a primary contributor to the cytoskeletal dysfunction. Nevertheless, the Rho/ROCK signaling pathway has been repeatedly described as a key player in oligodendrocyte maturation and myelination: Increased activation has been linked to OPC differentiation failure and conversely, pathway inhibition by siRNA, statins, or the small molecule RIs H1152 and Y-27632 has been shown to improve OPC differentiation, survival, and myelin formation suggesting a potential remyelination-promoting effect [4, 5, 27, 31, 36]. Therefore, we investigated whether the inhibition of the RhoA signaling pathway is able to restore process outgrowth and the ensheathment of axon-mimicking structures in cultures of aSyn-expressing hOLs. We differentiated hOLs expressing aSyn or GFP and applied the RI Y-27632 (RI, 2 µM) for four days (Fig. 8a). RI Y-27632 is a selective inhibitor of the p160-ROCK pathway commonly used for short-term application in stem cell cultures to improve cellular survival. In line with previous reports [21, 54], short-term administration of RI did not result in cellular toxicity (Fig. 8a). While the proportion of O4 + hOLs was slightly increased after RI administration (Fig. 8a), their potential to develop into mature MBP expressing hOLs remained unaffected (Fig. 8b). Importantly, the administration of RI shifted the cellular phenotype of aSyn expressing hOLs toward a comparable proportion of immature compact oligodendrocytes in control hOLs cultures (Fig. 8b). To explore the rescue potential of ROCK inhibition, we cultured EF-SNCA- and EF-GFP-transduced hOLs on aligned PCL nanofibers and quantified the formation of MBP + segments after RI administration. Indeed, EF-SNCA-transduced hOLs extended more processes after ROCK inhibition and showed a significantly increased formation of MBP + segments along nanofibers (Fig. 8c), suggesting a rescue of aSyn-mediated effects on oligodendrocyte morphology and the ensheathment of axon-mimicking structures.

## Discussion

The profound myelin loss and widespread neurodegeneration in MSA have been correlated with areas of aSyn-containing GCIs [30]. However, the underlying cellular and molecular pathomechanisms driving progressive loss of myelin in MSA remain poorly understood. Here, we present evidence for an aSyn-mediated dysfunction in hiPSC-derived oligodendrocytes associated with an impaired process outgrowth and altered actin dynamics. By targeting the cytoskeleton, we demonstrate the potential to restore process outgrowth and the capacity to ensheath axon-mimicking structures in vitro implying a novel therapeutic target in MSA.

In this study, we make use of a rapid and robust differentiation method to generate hOLs from hiPSCs, both, to dissect the role of aSyn in oligodendroglial maturation and the possibility of restoring aSyn-mediated oligodendroglial dysfunction. Within 20 days, we achieve a high yield of hOLs with over 68% of cells expressing O4, among which over 80% co-express MBP. The potential for oligodendroglial differentiation using the present forward programming by forced expression of SOX10, OLIG2, and NKX6.2 via NPCs is in the range achieved by single SOX10 [14] or combined SOX10/OLIG2 [34] expression in hiPSCs. Culturing these cells on aligned PCL nanofibers mimicking axon-like structures, the outgrowth of numerous MBP + processes was observed with the generation of multiple MBP + segments along nanofibers. To model increased intraoligodendroglial aSyn levels, hOLs were transduced with a lentiviral vector system expressing human aSyn. Despite robust expression of human aSyn in hOLs, there was no evidence of cellular toxicity, consistent with previous *post-mortem* findings in MSA and its corresponding transgenic animal model showing a preserved or even increased density of oligodendrocyte lineage cells in white matter regions, including striae within the basal ganglia or the corpus callosum [1, 11]. The current in vitro findings, together with previous *post-mortem* observations, suggest that the marked myelin deficit observed in MSA may be related to dysfunction of oligodendroglial cells rather than a net loss of oligodendrocytes. Consistently, decreased expression levels of proteins critical for differentiation and myelination have been reported in primary rodent cultures upon ectopic aSyn expression [10, 11], as well as in hiPSC-derived oligodendrocytes from MSA patients [3], suggesting impaired or delayed maturation into myelinating oligodendrocytes. Our investigation did not reveal any changes in the differentiation of NPCs into O4 + pre-myelinating oligodendrocytes or their subsequent maturation into MBP + oligodendrocytes, which contradicts the initial findings. However, considering that previous approaches were either based on primary OPCs or involved early OPCs as an intermediate differentiation stage, we speculate that the transcription factor-mediated differentiation of NPCs into pre-myelinating oligodendrocytes may have bypassed a vulnerable phase during early OPC differentiation. Importantly, myelination extends beyond developmental stages, with continuous myelin turnover and adaptive myelination persisting throughout adulthood [16, 26, 46, 63, 64]. In mice, these processes are mainly mediated by newly generated oligodendrocytes derived from resident OPCs [16, 26, 64], however, radiocarbon dating of oligodendrocytes in human brain tissue revealed a low rate of oligodendrocyte turnover [63], suggesting that myelin plasticity in the adult human brain relies primarily on mature or pre-myelinogenic oligodendrocytes, which are the main populations obtained by the present differentiation protocol.

Despite no obvious changes in hOL differentiation, the present model demonstrated a significant redistribution of TPPP within hOLs upon ectopic aSyn overexpression, with a significant increase in perinuclear accumulation. This observation is consistent with findings in human MSA tissue, where TPPP retracts from the myelin sheath and accumulates in the perinuclear cytoplasm as an early pathological event [29]. Our results, therefore, indicate a disease-relevant subcellular relocalization of TPPP in response to introligodendroglially elevated aSyn levels, despite unchanged total TPPP protein levels. Given prior evidence that TPPP facilitates aSyn aggregation [25], these findings support the notion of a bidirectional interplay between TPPP and aSyn. This further underscores the relevance of the present hOL model for investigating early molecular events, such as the TPPP-aSyn interplay, as a potential driver of oligodendroglial pathology in MSA. Consequently, future therapeutic strategies may aim to modulate protein redistribution and aggregation dynamics.

Dynamic morphological changes are a fundamental requirement for CNS myelination and myelin turnover [28, 66] and disruptions in myelin formation by aSyn may consequently lead not only to deficits in myelination but also to inadequate myelin preservation, potentially culminating in axonal and neuronal degeneration. In MSA, there is profound myelin loss associated with widespread neurodegeneration. However, the precise pathomechanisms linking both events remain elusive. A contribution of demyelination to neurodegeneration has previously been demonstrated in the cuprizone-induced demyelinating mouse model exhibiting severe axonal degeneration, which is preceded by profound myelin loss [61]. As myelin provides metabolic and trophic support for neurons at the axon-myelin interface (reviewed in [13, 37, 55]), demyelination might therefore deprive neurons of essential metabolites required to fulfill the energy demands for axonal transport, signal transduction, and homeostasis, potentially leading to degeneration. In line with the neuropathology observed in MSA patients, a significant demyelination and neuronal loss was present in the brain of a transgenic mouse model for MSA overexpressing aSyn in oligodendrocytes. Intriguingly, this loss was prevented by remyelination following the administration of the compound benztropine suggesting that myelin regeneration supports axonal integrity and survival, although a direct effect of the compound on neurons could not be excluded [10]. Given the critical role of oligodendrocytes and myelin in supplying energy and metabolites to neurons and its axons, further research should explore whether the aSyn-induced demyelination affects the metabolic coupling and thereby may contribute to a severe secondary neurodegeneration in MSA.

An increased proportion of compact oligodendrocytes with a large cell body together with a reduced number of primary processes and a decreased formation of MBP + segments along nanofibers were observed upon aSyn expression in hOLs, providing, to the best of our knowledge, the first evidence for an aSyn-mediated perturbation of oligodendrocyte morphology, and myelination in a human iPSC-derived in vitro model. By mimicking axonal structures in the absence of neurons, this model enables the investigation of intrinsically driven early events in myelination, particularly process outgrowth and ensheathment—both of which are critical prerequisites for myelin maintenance. However, it does not fully recapitulate the entire process of myelin formation, including the development of multi-layered sheaths and subsequent compaction. To explore the impact of aSyn overexpression on the ability of hOLs to ensheath neurites in a more physiological environment, we established a human triple-culture consisting of hOLs, hiPSC-derived cortical neurons, and human primary astrocytes. Consistent with our in vitro findings from the nanofiber assay, aSyn overexpression significantly impaired the overall capacity of hOLs to ensheath neurites. While the proportion of hOLs successfully ensheathing neurites was markedly reduced, we observed comparable or even enhanced formation of MBP + segments among hOLs with ensheathing events suggesting potential compensatory mechanisms. Previously, we reported an impaired process outgrowth and myelination deficit in rodent primary and stem cell derived cultures upon ectopic aSyn expression, which is consistent with our current observations [10, 11]. However, none of the previous studies focused on cytoskeletal and morphological changes. Interestingly, altered shape and oligodendroglial swelling are present in MSA brain tissue [53], resembling the current human aSyn-expressing hOL phenotype. The dynamic transformation during oligodendroglial maturation, such as process extension or myelin sheath formation, is controlled by dynamic processes of the actin cytoskeleton (reviewed in [57]). Increased fluorophore-coupled phalloidin signal intensity in aSyn-expressing hOLs indicated an increased level of actin filaments. Importantly, an increased putaminal level of actin filaments was confirmed in *post-mortem* putaminal tissue from MSA patients. Overall, our observations in both cultured hOLs upon aSyn overexpression and *post-mortem* MSA tissue underscore altered intraoligodendroglial actin cytoskeleton by promoting filament assembly. Since proper actin disassembly is essential for oligodendrocyte process outgrowth and myelin wrapping [66], aSyn-mediated alterations in actin reorganization are likely to impair oligodendroglial function, even in the absence of overt cell loss.

The dynamic changes of the actin cytoskeleton within oligodendrocytes are mediated by several actin regulators, including ERMIN, which plays an important role in cytoskeletal and myelin remodeling [6, 59]. In Ermin knock out mice, abnormal myelin architecture including loss of myelin segments and breakdown of myelinated fibers has been reported [59]. We observed a 60% downregulation of ERMIN mRNA in hOLs upon aSyn expression. Interestingly, this finding matches a previous report of reduced ERMIN transcripts in MSA oligodendrocytes obtained by laser capture microdissection from the cerebellar white matter of *post-mortem* brains [39]. However, bulk mRNA and protein analyses of *post-mortem* putamen tissue did not reveal significant changes in ERMIN at either level in the present study. Thus, the discrepancy between the previous study [39] using oligodendrocytes laser-captured from MSA brains and bulk *post-mortem* MSA putamen tissue in the present study may reflect the fact that ERMIN is an oligodendrocyte-specific protein, whereas bulk putamen tissue consists of a large diversity of neural cell types, potentially diluting oligodendrocyte-specific changes. Notably, despite the profound transcriptional repression of ERMIN in hOLs upon aSyn overexpression, protein levels remain unaffected. This may suggest the presence of compensatory mechanisms that stabilize ERMIN protein levels despite transcript reduction, for example through reduced protein turnover or other post-transcriptional regulations. Another possibility relates to the functional link between ERMIN and the actin cytoskeleton: ERMIN interacts with actin filaments and promotes cytoskeletal remodeling during oligodendrocyte process extension. The present hOL system, which shows increased F-actin assembly in response to aSyn overexpression, may counterbalance reduced ERMIN transcripts by reinforcing the ERMIN protein and prolonging its half-life through integration into a more stabilized actin cytoskeletal organization. This scenario would reconcile the transcript–protein mismatch and further support the notion that aSyn-induced cytoskeletal alterations impact ERMIN regulation at multiple levels, which warrants further investigation.

Accumulating evidence points to the Rho/ROCK signaling pathway as a key player in cytoskeletal remodeling, oligodendrocyte maturation and myelination [17, 31, 36]. Thus, we applied a small RI to aSyn-expressing hOLs to modulate process outgrowth and ensheathment. Indeed, ROCK inhibition resulted in an increased number of oligodendroglial processes and enhanced formation of MBP + segments of aSyn-expressing hOLs along nanofibers. Consistent with previous reports of a ROCK inhibition-mediated increased branching and accelerated myelin protein production in cellular cultures of rodent and human OPCs [36], our findings highlight the ROCK signaling pathway as a potential interventional target in MSA. Neuroprotective and remyelination-promoting therapies have attracted increased attention in recent years as therapeutic approaches for demyelinating diseases such as multiple sclerosis and MSA. Notably, the application of the RI Y-27632 resulted in increased myelination of lesioned areas in an in vitro model of spinal cord injury [5] and the RI fasudil is being investigated for its potential in the treatment of amyotrophic lateral sclerosis due to its promoting effect on neurite outgrowth and regeneration (https://clinicaltrials.gov/study/NCT03792490, Inhibition of Rho Kinase (ROCK) With Fasudil as Disease-modifying Treatment for ALS, Accessed 28 Jan 2025), further supporting the potential therapeutic value of ROCK inhibition in MSA. In conclusion, our findings provide evidence for a novel pathogenic cascade in MSA and highlight the potential of targeting the actin cytoskeleton and the ROCK signaling pathway as therapeutic strategies to promote remyelination in MSA.

## Supplementary Information

Below is the link to the electronic supplementary material.

Supplementary file1 (PDF 816 KB)

## Data Availability

All data supporting the results of this study is provided within the manuscript and its supplementary information.
