# Supplementary material for: Alpha synuclein-mediated cytoskeletal dysfunction impairs myelination in human oligodendrocytes
Source: Acta Neuropathol. 2025 Sep 19;150(1):33. doi: 10.1007/s00401-025-02933-z (PMC12449394; doi:10.1007/s00401-025-02933-z)
Supplement: Supplementary file 1 — Supplementary file1 (PDF 816 KB) [file 401_2025_2933_MOESM1_ESM.pdf]

Alpha synuclein–mediated cytoskeletal dysfunction impairs myelination in human oligodendrocytes

Jeanette Wihan[1,2], Kristina Battis[1], Alana Hoffmann[1,3,4], Farina Windener[5], Marcus Himmler[6], Anish Varghese[1], Aron Koller[1], Isabell Karnatz[2,7], Dirk W. Schubert[6], Friederike Zunke[1], Wei Xiang[1], Tanja Kuhlmann[5#] & Jürgen Winkler[1#]

[1] Division of Molecular Neurology, University Hospital Erlangen, Friedrich-Alexander-University (FAU) Erlangen-Nürnberg, 91054 Erlangen, Germany.

[2] Project Center for Stem Cell Process Engineering, Fraunhofer Institute for Biomedical Engineering (IBMT), 97070 Wuerzburg, Germany.

[3]Keenan Research Centre for Biomedical Science and Barlo Multiple Sclerosis Centre, St Michael's Hospital, Toronto, Ontario Canada

[4]Department of Immunology, The University of Toronto, Toronto, Ontario Canada

[5] Institute of Neuropathology, University Hospital Münster, 48149 Muenster, Germany.

[6] Institute of Polymer Materials, Department of Materials Science and Engineering, Friedrich-Alexander-University (FAU) Erlangen-Nürnberg, 91058, Erlangen, Germany.

[7] Department of Molecular and Cellular Biotechnology, Saarland University, 66123 Saarbrücken, Germany

# These authors jointly supervised this work: Tanja Kuhlmann & Jürgen Winkler

Corresponding authors

Jürgen Winkler, juergen.winkler@uk-erlangen.de

Jeanette Wihan, jeanette.wihan@ibmt.fraunhofer.de

Supplementary figures

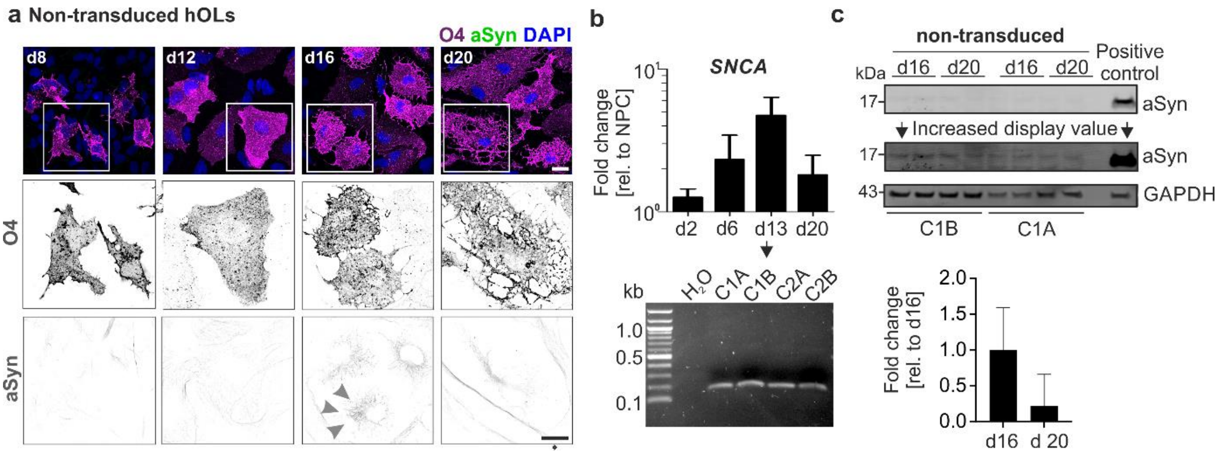

Suppl. Fig 1 Basal expression of endogenous alpha-synuclein (aSyn) in human oligodendrocytes (hOLs) during differentiation. (a) Representative immunocytochemistry images of endogenous aSyn expression in differentiating hOLs. Arrowheads indicate perinuclear aSyn localization in hOLs differentiated for 16 days. Scale bars: 20 µm. (b) Gene expression analysis of *SNCA* in hOLs from four hiPSC clones derived from two individuals. RT-qPCR products were analyzed by gel electrophoresis (bottom) after 13 days of differentiation, confirming the presence of transcripts. For quantification, mean values of three independent differentiation experiments were included for each hOL line (n = 4). (c) Western blot analysis of endogenous aSyn in hOL lines C1A and C1B, each measured in duplicates. Positive control: hOLs transduced with EF-SNCA and differentiated for 16 days. GAPDH was used as protein loading control. aSyn protein bands are visible after enhanced display settings. Together, immunocytochemistry, gene expression analysis, and Western blot analysis confirmed the presence of aSyn transcripts and protein in differentiating hOLs, albeit at very low levels, with a transient upregulation observed between days 13–16 of differentiation. Bar graphs represent mean + SD.

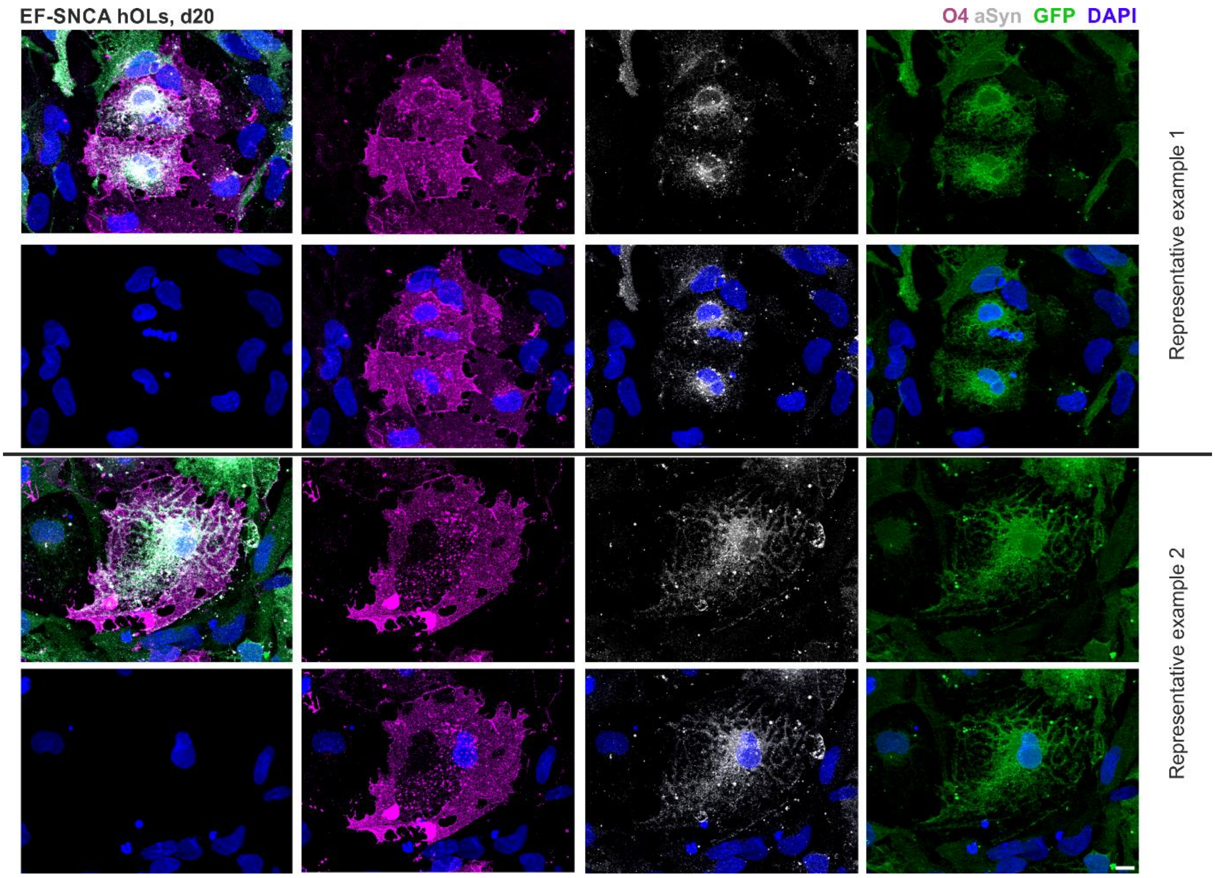

Suppl. Fig 2 Expression pattern of aSyn in EF-SNCA transduced hOLs. Representative example images of aSyn expression in hOLs at day 20 of differentiation. aSyn is primarily detected in the cytoplasm of transduced hOLs with a predominant enrichment in the perinuclear region. Scale bar: 10 µm.

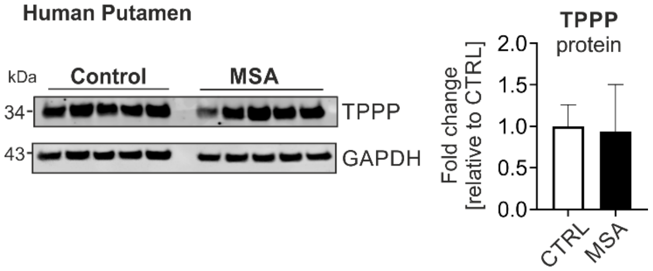

Suppl. Fig 3 TPPP levels in *post-mortem* putaminal tissue. TPPP protein levels in *post-mortem* putaminal tissue of MSA patients and controls. Mann-Whitney *U* test, $p = 0.8413$, n = 5. GAPDH served as loading control for Western blot analysis. Bar graphs represent the mean + SD.

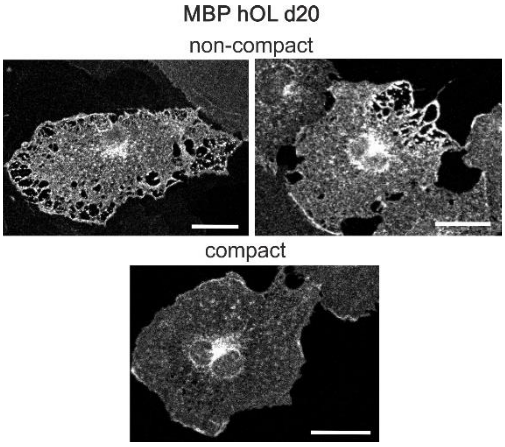

Suppl. Fig. 4 Visualization of compact and non-compact hOL subtypes using MBP immunostaining. Example images of MBP+ hOLs at day 20 of differentiation, with compact or non-compact morphology, are depicted. Scale bars: 20 µm

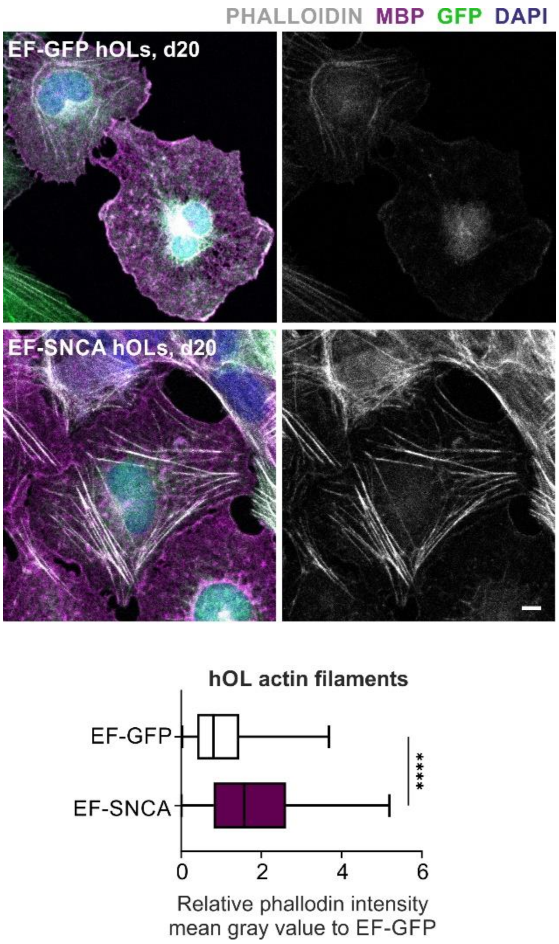

Suppl. Fig. 5 Effect of aSyn overexpression on actin filaments. Transduced hOLs were stained for MBP, actin filaments using fluorophore-coupled phalloidin, GFP, and nuclei (DAPI) at day 20 of differentiation. Quantification of actin filament levels at a single cell level based on phalloidin signal intensity. Student's t-test, p (phalloidin intensity) < 0.0001, n = 40 MBP + /GFP + cells of two hOL lines derived from the donor C1. Box plots show the 25. / 75. percentile; Whiskers of the box plots indicate the 5. / 95. percentile. Scale bar: 5 µm.

Supplementary tables

Supplementary table 1 Demographic and clinical characteristics of MSA patients and controls

Diagnosis	Analyzed regions	Sex	Age	PMD [h]	Brain weight [g]	Disease duration [months]
MSA*	putamen	f	66	08:05	1005	88
MSA*	putamen	f	67	07:15	1244	97
MSA	putamen	f	59	06:40	1102	54
MSA	putamen	m	55	08:40	1380	48
MSA	putamen	m	67	06:10	1376	77
Non-demented control	putamen	f	55	05:35	1363	
Non-demented control	putamen	f	60	07:30	1240	
Non-demented control	putamen	m	55	07:15	1393	
Non-demented control	putamen	m	51	07:45	1450	
Non-demented control	putamen	f	64	05:40	1221	

PMD, *post-mortem* delay; * donor tissue not used for phalloidin analysis

Supplementary table 2 List of oligonucleotides (forward and reverse primers) used for junction and quantitative polymerase chain reaction (qPCR).

Target		Primer sequence	Reference
18S rRNA	F<br>R	GGA GTA TGG TTG CAA AGC TGA<br>ATC TGT CAA TCC TGT CCG TGT	Viegas et al. 2017[5]
AAVS1, naïve	F<br>R	CTGTTTCCCCTTCCCAGGCAGGTCC<br>TGCAGGGGAACGGGGCTCAGTCTGA	Ghelman et al. 2021[3]
AAVS1, SON	F<br>R	CTGTTTCCCCTTCCCAGGCAGGTCC<br>TCGTCGCGGGTGGCGAGGCGCACCG	Ghelman et al. 2021[3]
ACTB	F<br>R	CCA ACC GCG AGA AGA TGA<br>TCC ATC ACG ATG CCA GTG	Hasegawa et al. 2010[4]
ARPC	F<br>R	AAC AGG AAG ATG AAG TGA TGA GAG<br>TTC ACA AAG CAA GTC CAC CAC	NM_001278556.2
CFL	F<br>R	CAA GAA GGC GGT CCT CT<br>ACA AAG GTG GCG TAG CG	NM_005507.3
ERMN	F<br>R	AGT CTG GAA GGT GCA CTC AC<br>GAG AGA AAG ATC TGT GAT AGC CT	NM_001009959.3
FSCN	F<br>R	CTGGCTACACGCTGGAGTTC<br>CTG AGTCCCCTGC TGTCTCC	Zhang et al. 2018[6]
GAPDH	F<br>R	GTC GGA GTC AAC GGA TTT G<br>TGG GTG GAATCA TAT TGG AAC	NM_002046.7
GSN	F<br>R	TTA CCG TGC AGC TGG ATG ACT ACC T<br>CGT GTT TGC CTG CTT GCC TTT C	NM_000177.5
MBP	F<br>R	TTA GCT GAA TTC GCG TGT GG<br>GAG GAA GTG AAT GAG CCG GTT A	Djelloul et al. 2015[1]

MOG	F<br>R	TGG CAA GCT TAT CAA GAC CCT C<br>CAC CTT TCC CTC ACC AAT AGC AT	NM_206809.4
PFL	F<br>R	GTGGAACGCCTACATCGACA<br>TGACCGGTCTTTGCCTACC	Zhao et al. 2020[7]
PLP	F<br>R	TGC TGA TGC CAG AAT GTA TGG<br>GCA GAT GGA CAG AAG GTT GGA	NM_000533.5
TPPP	F<br>R	GCT GCG TCT CCA AGC AAC AT<br>CAG TTC TTG CCG TGC ATC TC	NM_007030.3
VCL	F<br>R	CCAAAACATGTCTCCTATATCCTGG<br>GAAGTGTCCTTCAGACAGGG	NM_003373.4
WASL	F<br>R	AGTCCCTCTTCACTTTCCTC<br>GCTTTTCCCTTCTTCTTTTC	Frugtniet et al. 2017[2]

Supplementary table 3 List of primary antibodies used in this study

Antigen	Cat. No.	Company	Dilution
aSyn (15G7)	ALX-804-258-L001	Enzo Life Sciences	1: 500 (ICC)
aSyn (42)	610786	BD Biosciences	1: 500 (WB)
GAPDH	ab9485	Abcam	1: 1,000 (WB)
GAPDH	MAB374	Merck Millipore	1: 1,000 (WB)
GFAP	644702	Biolegend	1: 500 (ICC)
GFP	GFP-1020	Aves Labs	1: 500 (ICC)
ERMIN	orb183427	Biorbyt	1: 200 (WB)
Isotype IgM – APC (IS5 – 20C4)	130-093-176	Miltenyi Biotec	1: 50 (FC)
MBP	MCA409S	Bio – Rad	1: 500 (ICC)
NANOG	AF1997	R&D Systems	1: 300 (ICC)
NESTIN (10C2)	MAB5326	Merck Millipore	1: 300 (ICC)
O4	MAB1326	R&D Systems	1: 500 (ICC)
O4 IgM APC	130-095-891	Miltenyi Biotec	1: 50 (FC)
OCT 3 / 4 (C – 10)	sc-5279	Santa Cruz	1: 300 (ICC)
PLP	ab254363	Abcam	1: 500 (ICC)
SOX 2 (Y – 17)	sc-17320	Santa Cruz	1: 300 (ICC)
TPPP	PA5-19243	Thermo Fisher Scientific	1: 500 (WB)<br>1:100 (ICC)
TUBB3	802001	Biolegend	1: 500 (ICC)

FC, flow cytometry; ICC, immunocytochemistry; WB, western blot

References

1 Djelloul M, Holmqvist S, Boza-Serrano A, Azevedo C, Yeung MS, Goldwurm S, Frisen J, Deierborg T, Roybon L (2015) Alpha-Synuclein Expression in the Oligodendrocyte Lineage: an In Vitro and In Vivo Study Using Rodent and Human Models. Stem Cell Reports 5: 174-184 Doi 10.1016/j.stemcr.2015.07.002

2 Frugtniet BA, Martin TA, Zhang L, Jiang WG (2017) Neural Wiskott-Aldrich syndrome protein (nWASP) is implicated in human lung cancer invasion. BMC Cancer 17: 224 Doi 10.1186/s12885-017-3219-3

3 Ghelman J, Grewing L, Windener F, Albrecht S, Zarbock A, Kuhlmann T (2021) SKAP2 as a new regulator of oligodendroglial migration and myelin sheath formation. Glia 69: 2699-2716 Doi 10.1002/glia.24066

4 Hasegawa D, Fujii R, Yagishita N, Matsumoto N, Aratani S, Izumi T, Azakami K, Nakazawa M, Fujita H, Sato Tet al (2010) E3 ubiquitin ligase synoviolin is involved in liver fibrogenesis. PLoS One 5: e13590 Doi 10.1371/journal.pone.0013590

5 Viegas CSB, Costa RM, Santos L, Videira PA, Silva Z, Araujo N, Macedo AL, Matos AP, Vermeer C, Simes DC (2017) Gla-rich protein function as an anti-inflammatory agent in monocytes/macrophages: Implications for calcification-related chronic inflammatory diseases. PLoS One 12: e0177829 Doi 10.1371/journal.pone.0177829

6 Zhang H, Cong QX, Zhang SG, Zhai XW, Li HF, Li SQ (2018) High Expression Levels of Fascin-1 Protein in Human Gliomas and its Clinical Relevance. Open Med (Wars) 13: 544-550 Doi 10.1515/med-2018-0080

7 Zhao YF, He XX, Song ZF, Guo Y, Zhang YN, Yu HL, He ZX, Xiong WC, Guo W, Zhu XJ (2020) Human antigen R-regulated mRNA metabolism promotes the cell motility of migrating mouse neurons. Development 147: Doi 10.1242/dev.183509
